# Supplementary material for: A General Picture of Cucurbit[8]uril Host–Guest Binding: Recalibrating Bonded Interactions
Source: Molecules. 2023 Mar 31;28(7):3124. doi: 10.3390/molecules28073124 (PMC10095826; doi:10.3390/molecules28073124)
Supplement: Supplementary file 1 [file molecules-28-03124-s001.zip › molecules-2241132-supplementary.pdf]

Supporting Information: A General Picture of Cucurbit[8]uril Host-Guest Binding: Recalibrating Bonded Interactions

Zhaoxi Sun [1,*], Qiaole He [2], Zhihao Gong [3,4], Payam Kalhor [5], Zhe Huai [6,*], Zhirong Liu [1]

[1]*College of Chemistry and Molecular Engineering, Peking University, Beijing 100871, China*

[2]*AI Department of Enzymaster (Ningbo) Bio-Engineering Co., Ltd., North Century Avenue 333, 315100 Ningbo, China*

[3]*School of Micro-Nano Electronics, Zhejiang University, Hangzhou 310027, China*

[4]*Hangzhou Global Scientific and Technological Innovation Center, Zhejiang University, Hangzhou 310027, China*

[5] *Institute of Nanotechnology, Karlsruhe Institute of Technology, Hermann-von-Helmholtz-Platz 1, 76344 Eggenstein-Leopoldshafen, Germany*

[6] *XtalPi - AI Research Center (XARC), 7F, Tower A, Dongsheng Building, No.8, Zhongguancun East Road, Haidian District, Beijing 100083, China*

*To whom correspondence should be addressed: z.sun@pku.edu.cn (Z.S.), zhe.huai@xtalpi.com (Z.H.)

S1. Detailed discussions about the radius-contact surfaces.

We start by checking the GAFF2 results. The radius-contact ($\rho - C$) surfaces under this force field are presented in Fig. S9. For the guest G1, only one wide free energy minimum is observed. The bound conformation extracted there features the whole guest fluctuating inside the host cavity and the formation of inter-molecular -NH_2^+ ··· O=C- hydrogen bonds, which is similar to the GAFF case observed in our previous work.[92] As for the structurally complex guest G2, there are three bound conformations under GAFF2. The two small-C minima with higher thermodynamic stabilities feature the phenyl ring connected to the amide bond staying at the center of the host and the extended guest clinging to one side of the host, and their differences mainly lie in the degree of distortion to the CB8 ring. The other large-C minimum is less stable, and its larger host-guest contact number arises from the fact that the middle part of the guest is coordinated at the host center and the two phenyl rings are outside the cavity (i.e., solvent-exposed). All these conformations are observed and of high thermodynamic stabilities under GAFF. Thus, the patterns of the host-guest coordination are not significantly different under the GAFF derivatives. For the structurally rigid guest G3, similar to the GAFF case, we only observe one narrow free energy minimum, the structural features of which include the center-binding behavior and the inter-molecular hydrogen bonds formed between the -NH^+ and the two -OH groups of the guest and the -C=O portals of the host. The free energy surface for the CB8-G4 complex is altered compared with the previous GAFF result. The bound state becomes less stable when replacing GAFF with GAFF2, and the host-guest interaction is weaker. The most stable bound conformation locates at the largest-C minimum, which depicts a center-binding pose with -NH^+ and -OH of the guest forming hydrogen bonds with the -C=O portals of the host. When the host-guest COM distance grows, the guest gradually leaves the cavity center. There are two wide free energy minima along the dissociation pathway. In the first minimum with larger host-guest contacts, the guest starts to leave the host center but the inter-molecular hydrogen bonds are still formed between -NH^+ and -OH and the -C=O portals. When the host-guest COM distance further increases, the system reaches the second minimum, where only the -NH^+ ··· -C=O hydrogen bond remains. The protonated and deprotonated forms of G5 have similar radius-contact surfaces. Center-binding poses with the whole guest fluctuating inside the host cavity are observed in their bound conformations, which is still similar to the GAFF case. The situation is similar for the guest G6, where center-binding poses with many host-guest coordination patterns are observed. As for the last guest G7, still many interaction patterns could be observed. For instance, in the center-binding pose, the phenyl ring could be in or outside the central cavity. Therefore, the radius-contact surfaces obtained under GAFF2 are similar to the GAFF results.

We then turn to the first refitted parameter set FM-PM6. The radius-contact surfaces are presented in Fig. S10. For the structurally simple guest G1, the bound conformation still features the guest fluctuating inside the host

cavity. However, compared with the GAFF2 case, the host cavity is more flexible. The rectangular host cavity observed in the unbound state (c.f., Fig. 4e) is still thermodynamically stable in the bound state, but many other shapes are also favorable. The presence of these irregularly shaped host rings suggests that the inter-molecular host-guest interactions could alter the dynamic behavior of the host, and insights obtained in host-only or guest-only simulations could be limited. Note that the change of the bonded parameters for the guest G1 also plays a role in the alteration of host-guest binding. The free energy surface of the structurally complex G2 under FM-PM6 also shows some differences compared with GAFF2. There are one wide large-distance and one narrow small-distance minima, both of which are center-binding poses that have been observed under GAFF2. The two narrow large-distance minima under GAFF2 are merged into the wide large-distance minimum under FM-PM6, and the FM-PM6 minimum becomes further wider due to the increased flexibility of the host ring under this parameter set. As for the small-distance minimum under GAFF2, it becomes thermodynamically stable under FM-PM6 also due to the increased flexibility of the host ring. The wide large-distance minimum contains many host-guest coordination patterns. When comparing the bound conformations extracted from the small-distance minimum and the region with similar host-guest contacts in the large-distance minimum, some structural features unique in each conformational state could be identified. For instance, two -CH_2- groups of the host rotate into the inner side of the cavity but show different behaviors in the two conformational states. In the large-distance minimum, these two groups are symmetric on the host ring, i.e., in the ith and (i+4)th repeating units, which leads to a relatively symmetric host cavity. As the guest is clinging to one side of the host (i.e., asymmetric binding), the COMs of the host and the guest are separated relatively far. By contrast, in the small-distance minimum, the two -CH_2- groups are distributed on the ith and (i+3)th repeating units, which leads to a severely asymmetric host cavity. The asymmetric binding of the guest to the host ring and the asymmetric orientation of the -CH_2- groups leads to tighter host-guest coordinations and thus a smaller COM distance. Similar to the cases of the GAFF derivatives, the radius-contact free energy surface for the structurally rigid G3 under FM-PM6 only has one narrow free energy minimum, which suggests that the number of favorable host-guest coordination patterns is limited. In the center-binding pose, the -NH^+ and 2 -OH groups of the guest form inter-molecular hydrogen bonds with the -C=O portals of the host. However, the shape of the host cavity is quite irregular, which is in agreement with the increased host flexibility observed in unbiased simulations. Therefore, we can see that the bonded parameters could have significant impacts on the host-guest coordination pattern. As for the structurally rigid G4, two free energy minima are observed on the radius-contact surfaces, which is similar to the GAFF2 case. The first large-C center-binding pose features the -NH^+ and -OH groups of the guest forming inter-molecular hydrogen bonds with the -C=O portals of the irregularly shaped host, while the second large-distance minimum has some parts of the guest leaving the host cavity. As for the protonated G5, the only free

energy minimum observed is quite wide, which is different from the previous GAFF2 case. The inter-molecular hydrogen bond is still formed between the $-NH_2^+$ group of the guest and one of the -C=O portals of the host, and the guest fluctuates inside the host cavity in the center-binding mode. However, the host cavity is quite flexible under FM-PM6. The simultaneous conformational fluctuations of the irregularly shaped host and the guest make the host-guest coordination flexible. The situation is similar for the deprotonated G5. Namely, the narrow GAFF2 minimum becomes wider when shifting to FM-PM6, due to the increased flexibility of the host cavity. For the structurally complex G6, there is only one free energy minimum on the radius-contact surface. Similar to the GAFF2 result, this minimum is narrow and still features center-binding. As for the last guest G7, two free energy minima are observed on the radius-contact surface. The bound conformations extracted from these minima feature the center-binding behavior with many interaction patterns. For instance, the phenyl ring of the guest could stay in or outside the host cavity. The shape of the host is still irregular. When comparing the FM-PM6 surface with the GAFF2 one, we know that the wide minimum containing a variety of binding poses under GAFF2 is split into the two sub-minima under FM-PM6. The refitted FM-PM6 CB8 ring could alter its conformation to tightly coordinate the guest, while under GAFF2 the ring conformation remains almost unchanged. Note that the refitting of the bonded parameters of the guest also plays a role here. Overall, the FM-PM6 parameter set provides similar descriptions of host-guest coordination to the GAFF2 case, but the detailed interaction patterns differ in many aspects.

We finally investigate the last bonded parameter set FM-BLYP. The radius-contact surfaces under this parameter set are shown in Fig. S11. As shown in the unbiased simulations under different bonded parameter sets, the host behaviors under the FM-BLYP parameter set are similar to those under GAFF2. Thus, we expect the host conformation to be similar to the GAFF2 case in host-guest coordination. However, as the refitting procedure also alters the description of the guests, the conformational preference, the coordination pattern and the strength of inter-molecular interactions are expected to be altered. For the structurally simple guest G1, a wide free energy minimum is observed. The guest fluctuates inside the central cavity and forms inter-molecular hydrogen bonds with the -C=O portals of the host. Similar to GAFF2, the host ring is less distorted compared with the FM-PM6 case. As for the structurally complex G2, the host-guest binding pattern is still the extended guest clinging to one side of the host. The low-(free-)energy regions are similar to the previous cases. From these free energy minima, many binding poses could be observed. For example, the middle part of the guest can stay at the host center and its $-NH^+$ forms a hydrogen bond with one -C=O portal of the host. The G3 case is still similar to the previous cases. In the only free energy minimum, the guest binds to the center of the host with its $-NH^+$ and 2 -OH groups forming inter-molecular hydrogen bonds with the -C=O portals of the host. The structurally rigid G4 under FM-BLYP only has one free energy minimum, where the center-binding pose is observed and its $-NH^+$ and -OH groups form hydrogen bonds

with the -C=O portals of the host. This difference between the FM-BLYP and GAFF2 results mainly arises from the change of the intra-molecular interactions of the guest G4, which suggests that only monitoring the behavior of the host dynamics could lead to limited insights about inter-molecular coordination and the system-specific refitting of the bonded terms to improve the accuracy of the description is necessary even for structurally rigid guests. As for the protonated G5, only one free energy minimum is observed on the radius-contact surface. The FM-BLYP minimum is wider than the GAFF2 one but is narrower than the FM-PM6 case. The most stable bound conformation has the whole guest inside the host cavity, and conformational fluctuations would cause some parts of the guest to leave the host center and become solvent-exposed. The situation is similar for the deprotonated G5. Specifically, there is only one minimum observed on the radius-contact surface with the guest fluctuating inside the host cavity. Two free energy minima are observed for the guest G6. The large-C minimum is more thermodynamically stable than the other one. The guest binds at the center of the host, and conformational fluctuations would lead to some parts of the guest leaving the host cavity, producing less compact bound conformations. Note that this two-minima behavior differs from the GAFF2 and FM-PM6 results, which again suggests that although the GAFF2 and FM-BLYP parameter sets produce similar host dynamics, the alteration of the guest description would lead to obvious differences in inter-molecular coordination. A wide free energy minimum is observed for the last guest G7. This observation is similar to the GAFF2 case, but this wide minimum is cut into two narrower ones under FM-PM6. The wideness of this bound state indicates the existence of many host-guest binding patterns.

Fig. S1. The correlations between the MM and SQM (PM6-D3H4X) energetics calculated from 25 ns trajectories generated at 600 K (left) and 300 K (right) in vacuo for the host and 7 guest molecules. The sampling interval is 5 ps and there are 5000 independent configurations in total. The RMSE and MAE of the original parameter set and the newly fitted force-matching set are also presented. Note that the protonated and deprotonated Ketamine are fitted separately.

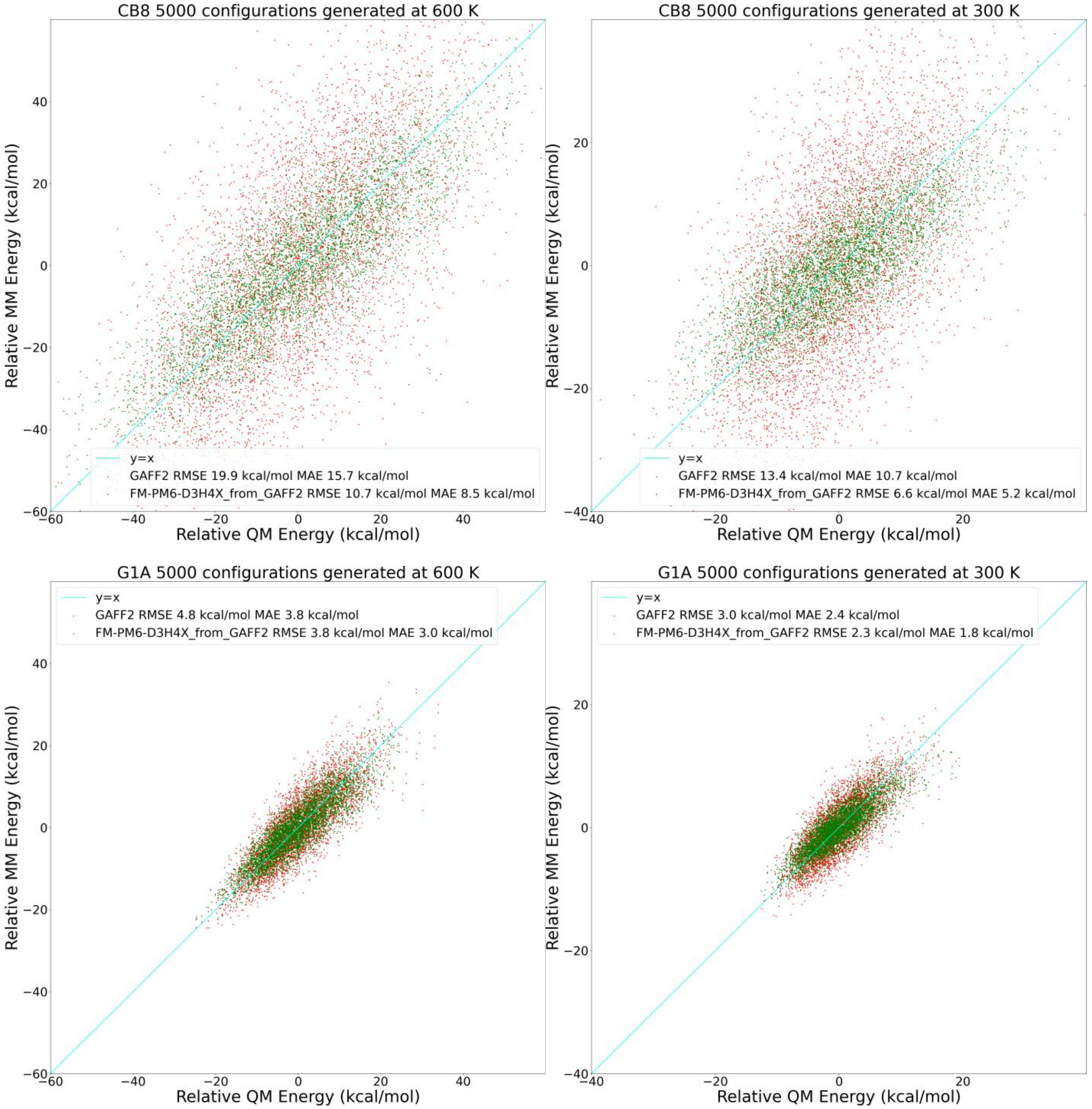

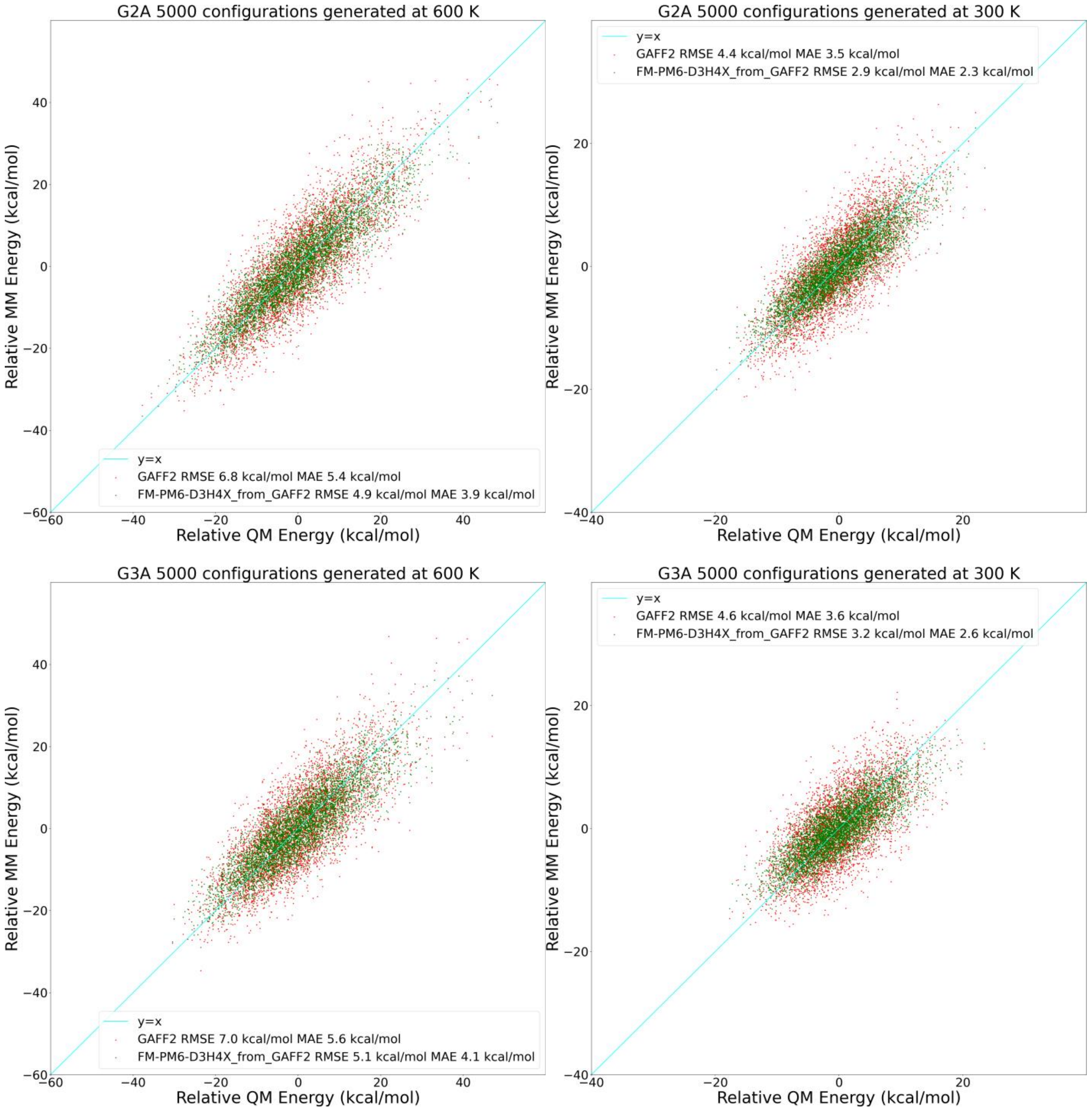
G2A 5000 configurations generated at 600 K
y=x
GAFF2 RMSE 6.8 kcal/mol MAE 5.4 kcal/mol
FM-PM6-D3H4X_from_GAFF2 RMSE 4.9 kcal/mol MAE 3.9 kcal/mol
G2A 5000 configurations generated at 300 K
y=x
GAFF2 RMSE 4.4 kcal/mol MAE 3.5 kcal/mol
FM-PM6-D3H4X_from_GAFF2 RMSE 2.9 kcal/mol MAE 2.3 kcal/mol
G3A 5000 configurations generated at 600 K
y=x
GAFF2 RMSE 7.0 kcal/mol MAE 5.6 kcal/mol
FM-PM6-D3H4X_from_GAFF2 RMSE 5.1 kcal/mol MAE 4.1 kcal/mol
G3A 5000 configurations generated at 300 K
y=x
GAFF2 RMSE 4.6 kcal/mol MAE 3.6 kcal/mol
FM-PM6-D3H4X_from_GAFF2 RMSE 3.2 kcal/mol MAE 2.6 kcal/mol
Relative MM Energy (kcal/mol)
Relative QM Energy (kcal/mol)

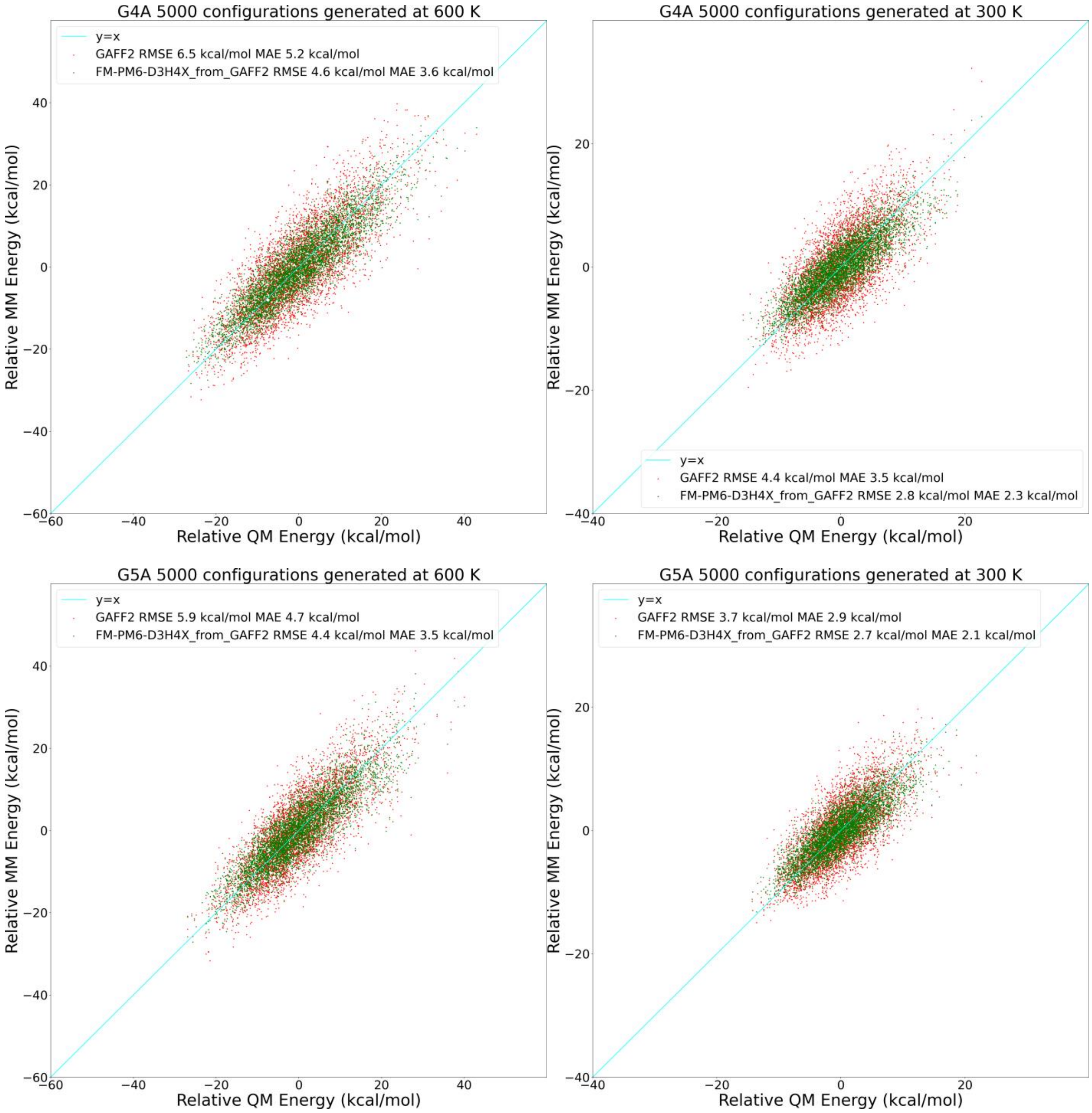
G4A 5000 configurations generated at 600 K
y=x
GAFF2 RMSE 6.5 kcal/mol MAE 5.2 kcal/mol
FM-PM6-D3H4X_from_GAFF2 RMSE 4.6 kcal/mol MAE 3.6 kcal/mol
Relative MM Energy (kcal/mol)
Relative QM Energy (kcal/mol)
G4A 5000 configurations generated at 300 K
y=x
GAFF2 RMSE 4.4 kcal/mol MAE 3.5 kcal/mol
FM-PM6-D3H4X_from_GAFF2 RMSE 2.8 kcal/mol MAE 2.3 kcal/mol
Relative MM Energy (kcal/mol)
Relative QM Energy (kcal/mol)
G5A 5000 configurations generated at 600 K
y=x
GAFF2 RMSE 5.9 kcal/mol MAE 4.7 kcal/mol
FM-PM6-D3H4X_from_GAFF2 RMSE 4.4 kcal/mol MAE 3.5 kcal/mol
Relative MM Energy (kcal/mol)
Relative QM Energy (kcal/mol)
G5A 5000 configurations generated at 300 K
y=x
GAFF2 RMSE 3.7 kcal/mol MAE 2.9 kcal/mol
FM-PM6-D3H4X_from_GAFF2 RMSE 2.7 kcal/mol MAE 2.1 kcal/mol
Relative MM Energy (kcal/mol)
Relative QM Energy (kcal/mol)

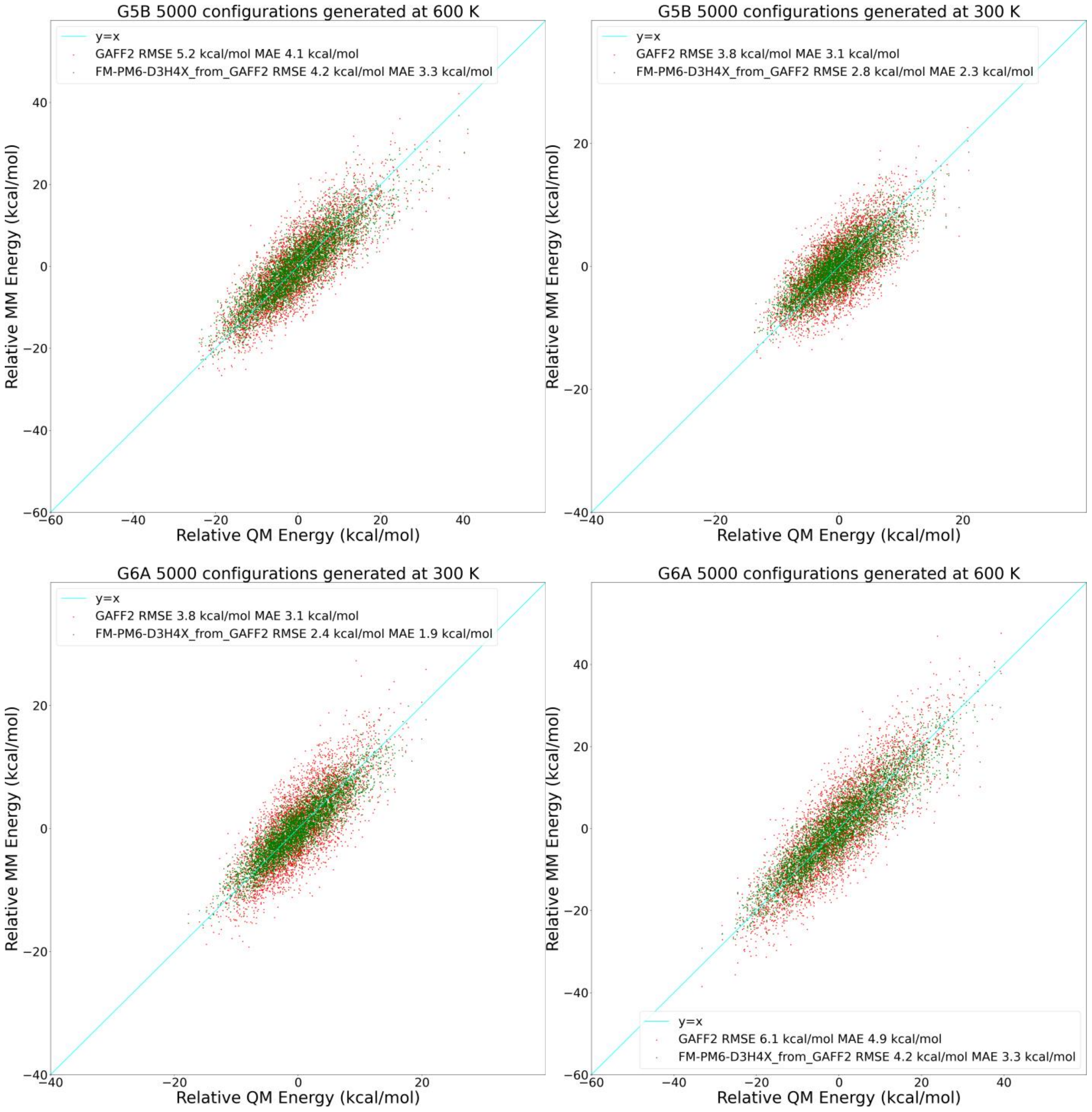
G5B 5000 configurations generated at 600 K
y=x
GAFF2 RMSE 5.2 kcal/mol MAE 4.1 kcal/mol
FM-PM6-D3H4X_from_GAFF2 RMSE 4.2 kcal/mol MAE 3.3 kcal/mol
Relative MM Energy (kcal/mol)
Relative QM Energy (kcal/mol)
G5B 5000 configurations generated at 300 K
y=x
GAFF2 RMSE 3.8 kcal/mol MAE 3.1 kcal/mol
FM-PM6-D3H4X_from_GAFF2 RMSE 2.8 kcal/mol MAE 2.3 kcal/mol
Relative MM Energy (kcal/mol)
Relative QM Energy (kcal/mol)
G6A 5000 configurations generated at 300 K
y=x
GAFF2 RMSE 3.8 kcal/mol MAE 3.1 kcal/mol
FM-PM6-D3H4X_from_GAFF2 RMSE 2.4 kcal/mol MAE 1.9 kcal/mol
Relative MM Energy (kcal/mol)
Relative QM Energy (kcal/mol)
G6A 5000 configurations generated at 600 K
y=x
GAFF2 RMSE 6.1 kcal/mol MAE 4.9 kcal/mol
FM-PM6-D3H4X_from_GAFF2 RMSE 4.2 kcal/mol MAE 3.3 kcal/mol
Relative MM Energy (kcal/mol)
Relative QM Energy (kcal/mol)

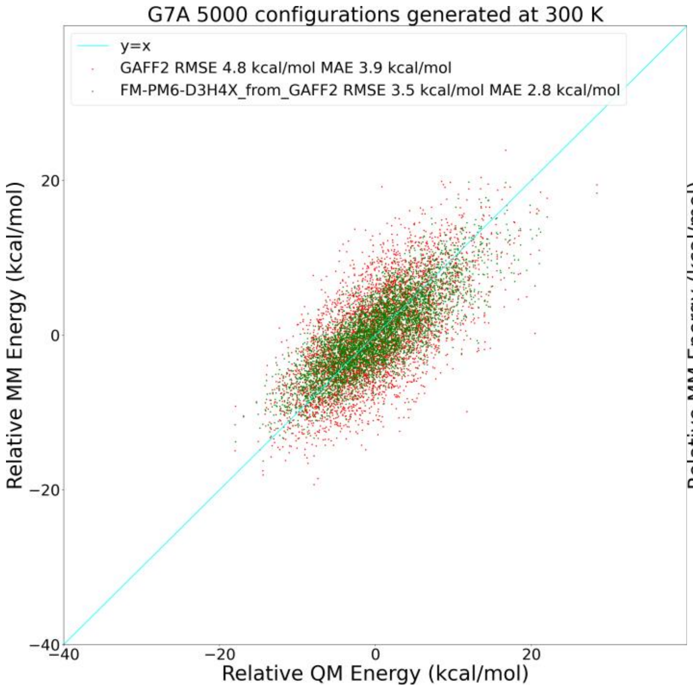

G7A 5000 configurations generated at 300 K
y=x
GAFF2 RMSE 4.8 kcal/mol MAE 3.9 kcal/mol
FM-PM6-D3H4X_from_GAFF2 RMSE 3.5 kcal/mol MAE 2.8 kcal/mol
Relative MM Energy (kcal/mol)
Relative QM Energy (kcal/mol)

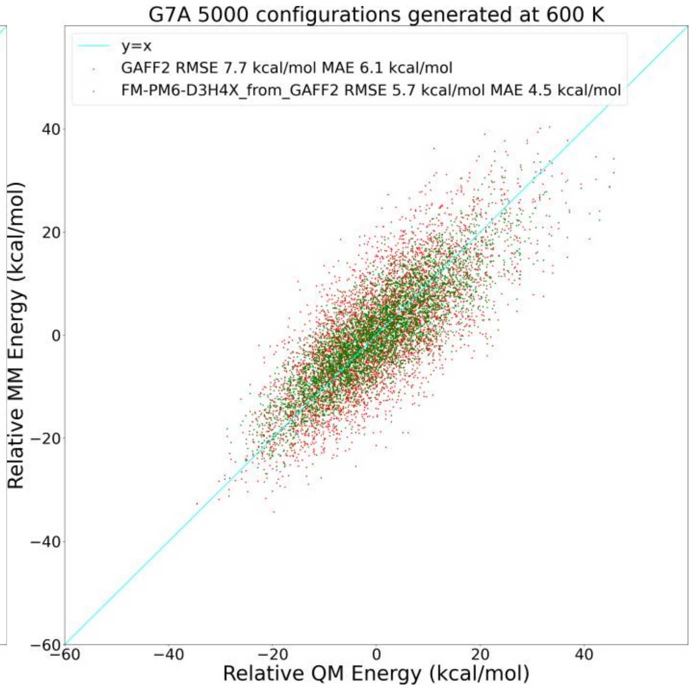

G7A 5000 configurations generated at 600 K
y=x
GAFF2 RMSE 7.7 kcal/mol MAE 6.1 kcal/mol
FM-PM6-D3H4X_from_GAFF2 RMSE 5.7 kcal/mol MAE 4.5 kcal/mol
Relative MM Energy (kcal/mol)
Relative QM Energy (kcal/mol)

Fig. S2. The time series of the errors of atomic forces ($\|\Delta \mathbf{F}_i\|_2$ for the *i*th atom) under the original GAFF2 and the refitted FM-PM6_from_GAFF2 parameter set calculated from 25 ns trajectories generated at 600 K and 300 K in vacuo for the host and 7 guest molecules. The protonated and deprotonated forms of Ketamine are parameterized and thus tested separately. The sampling interval is 5 ps and there are 5000 independent configurations in total. Red dots for the force errors larger than 50 kcal/(mol·Å), green for force errors larger than 30 kcal/(mol·Å), blue for errors larger than 10 kcal/(mol·Å), and white for the other small-error points. The overall RMSEs of atomic forces of each molecule in kcal/(mol·Å·atom) under the original GAFF2 and the newly obtained FM-PM6 parameter sets are also given.

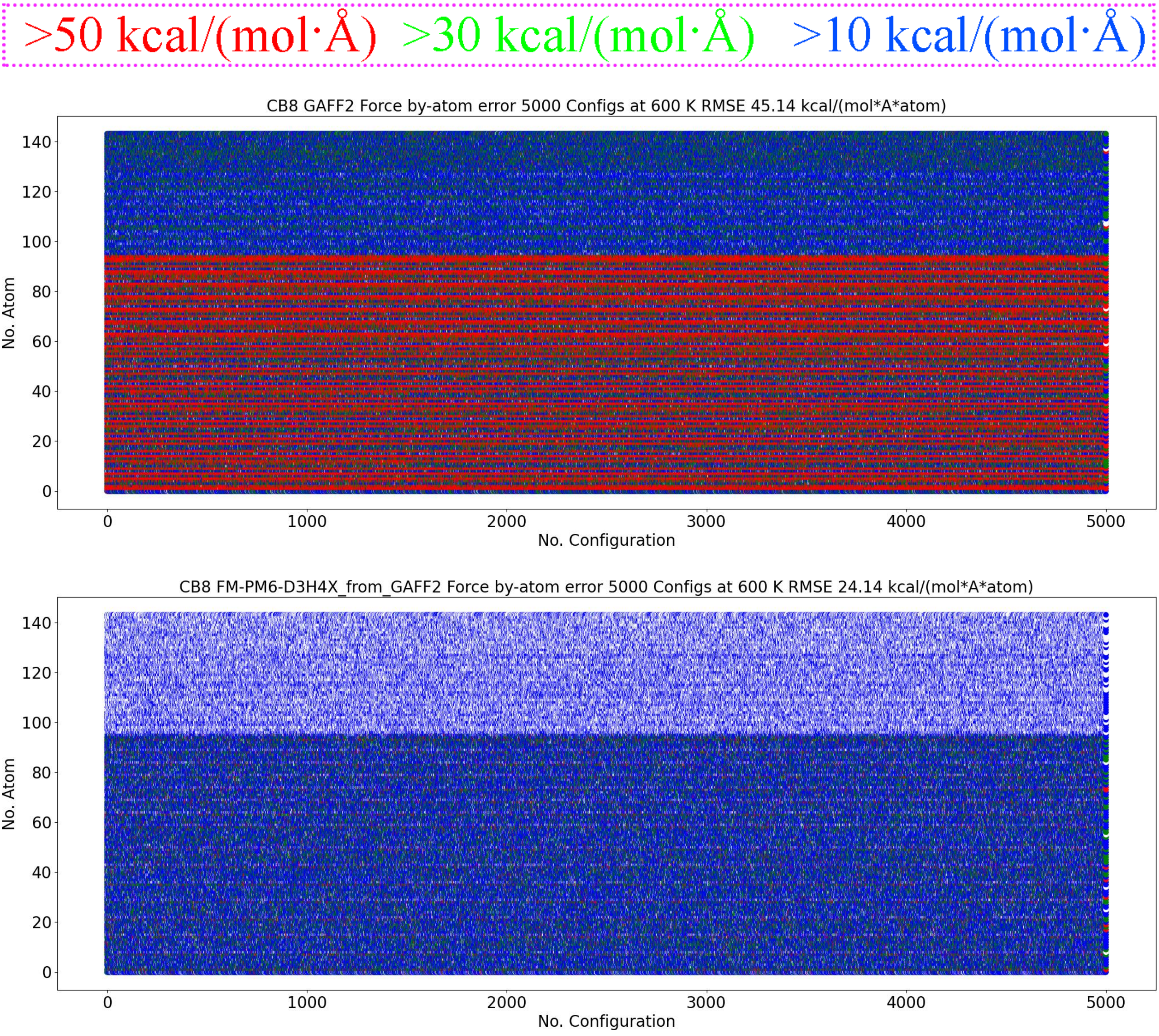

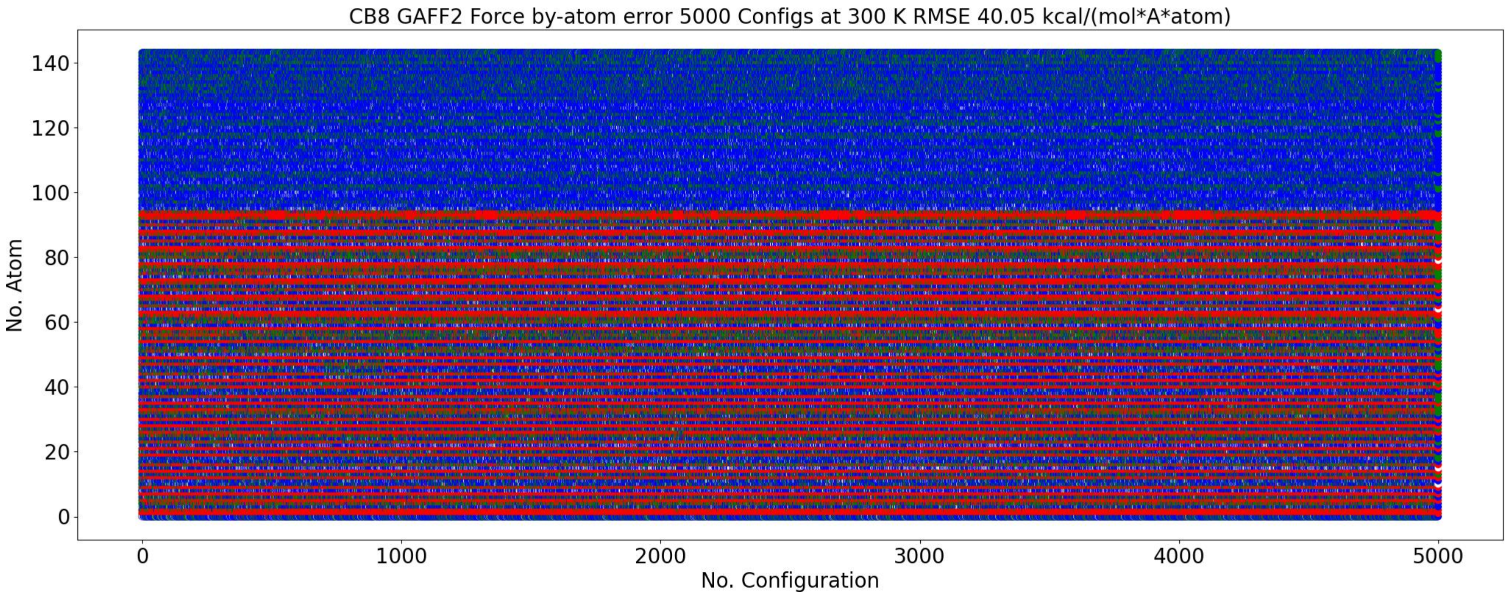
CB8 GAFF2 Force by-atom error 5000 Configs at 300 K RMSE 40.05 kcal/(mol*A*atom)
No. Atom
No. Configuration
0
20
40
60
80
100
120
140
0
1000
2000
3000
4000
5000

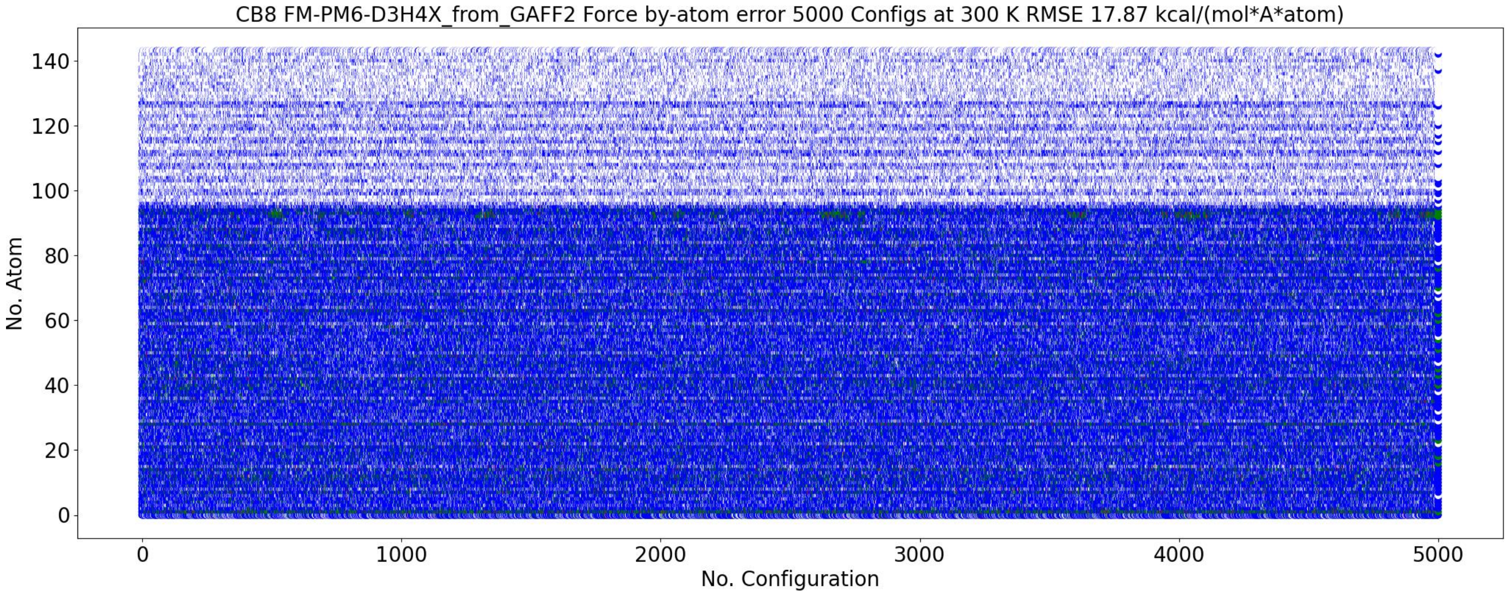
CB8 FM-PM6-D3H4X_from_GAFF2 Force by-atom error 5000 Configs at 300 K RMSE 17.87 kcal/(mol*A*atom)
No. Atom
No. Configuration
0
20
40
60
80
100
120
140
0
1000
2000
3000
4000
5000

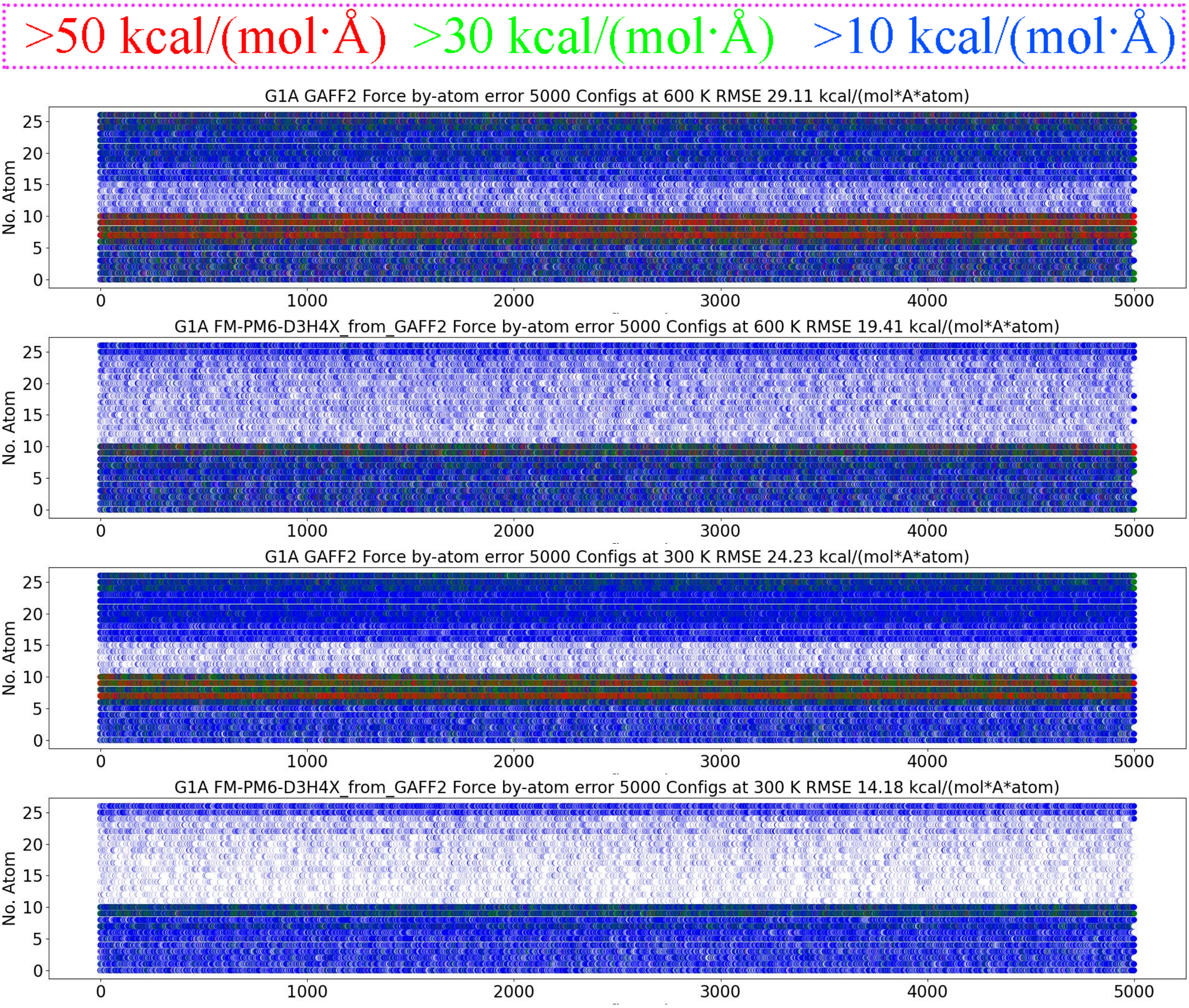
>50 kcal/(mol·Å) >30 kcal/(mol·Å) >10 kcal/(mol·Å)
G1A GAFF2 Force by-atom error 5000 Configs at 600 K RMSE 29.11 kcal/(mol*A*atom)
G1A FM-PM6-D3H4X_from_GAFF2 Force by-atom error 5000 Configs at 600 K RMSE 19.41 kcal/(mol*A*atom)
G1A GAFF2 Force by-atom error 5000 Configs at 300 K RMSE 24.23 kcal/(mol*A*atom)
G1A FM-PM6-D3H4X_from_GAFF2 Force by-atom error 5000 Configs at 300 K RMSE 14.18 kcal/(mol*A*atom)
No. Atom
0
5
10
15
20
25
1000
2000
3000
4000
5000

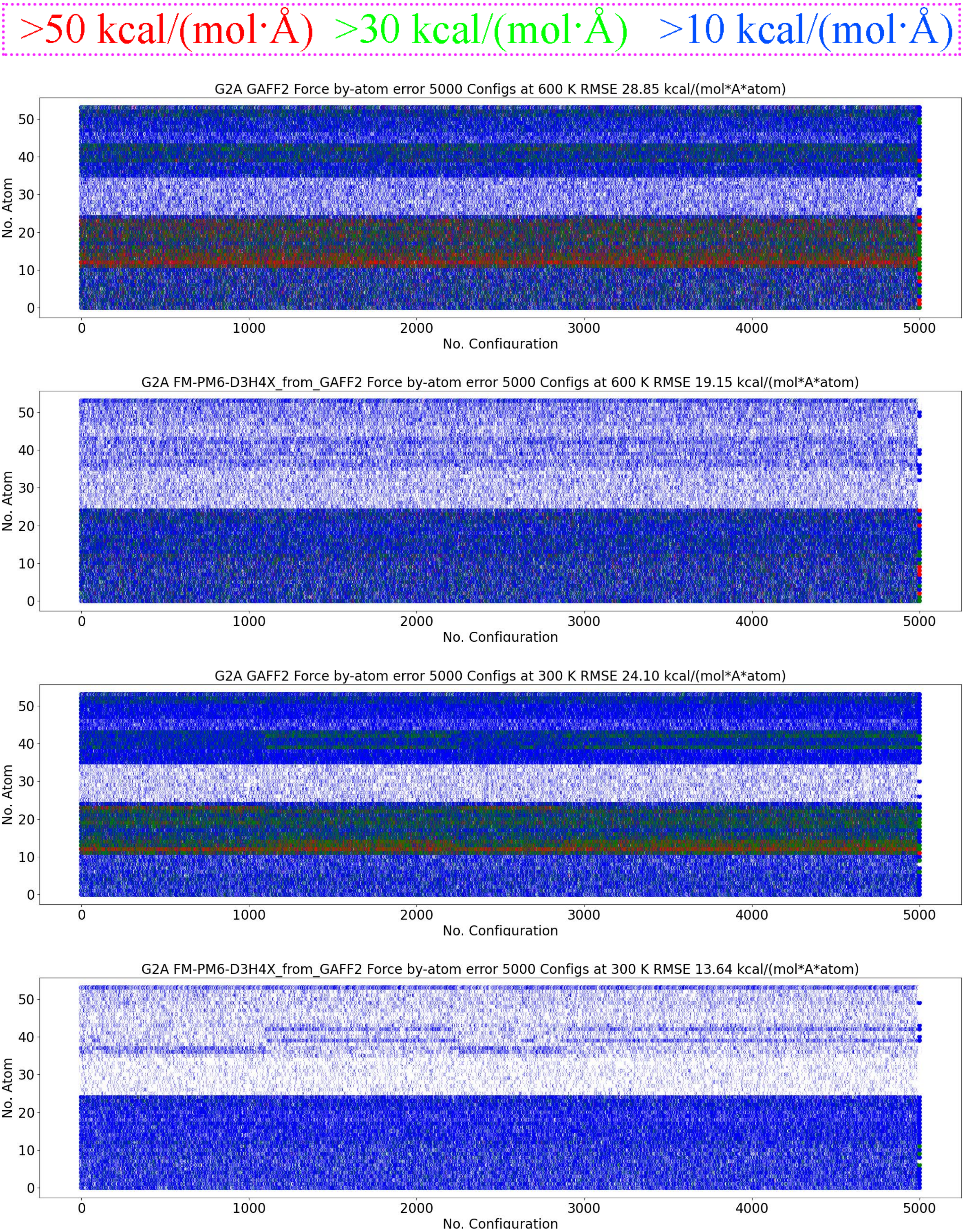
>50 kcal/(mol·Å)
>30 kcal/(mol·Å)
>10 kcal/(mol·Å)
G2A GAFF2 Force by-atom error 5000 Configs at 600 K RMSE 28.85 kcal/(mol*A*atom)
G2A FM-PM6-D3H4X_from_GAFF2 Force by-atom error 5000 Configs at 600 K RMSE 19.15 kcal/(mol*A*atom)
G2A GAFF2 Force by-atom error 5000 Configs at 300 K RMSE 24.10 kcal/(mol*A*atom)
G2A FM-PM6-D3H4X_from_GAFF2 Force by-atom error 5000 Configs at 300 K RMSE 13.64 kcal/(mol*A*atom)
No. Atom
No. Configuration

>50 kcal/(mol·Å) >30 kcal/(mol·Å) >10 kcal/(mol·Å)

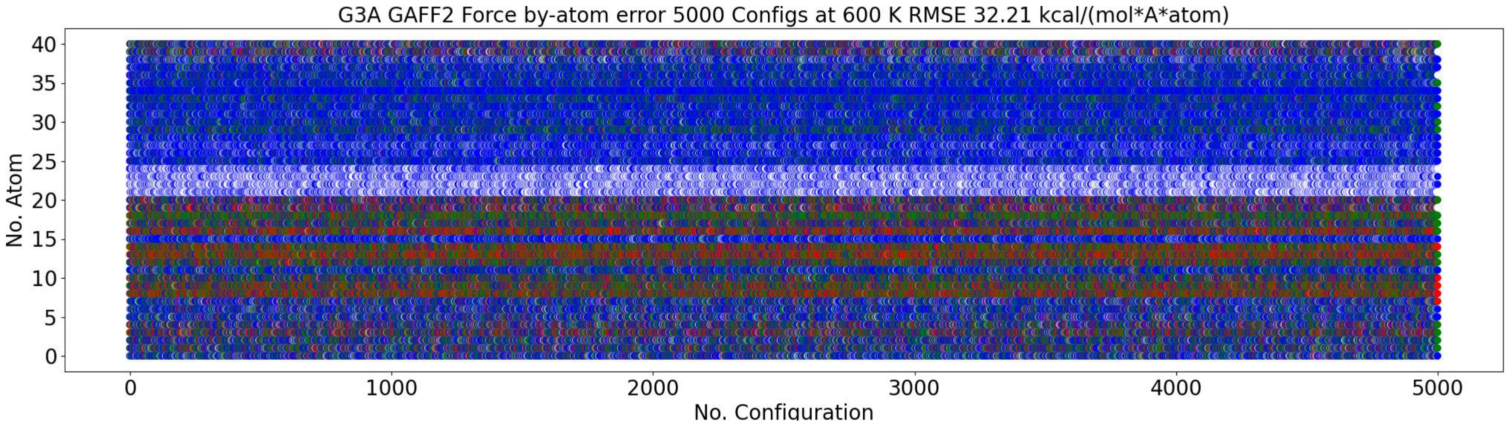

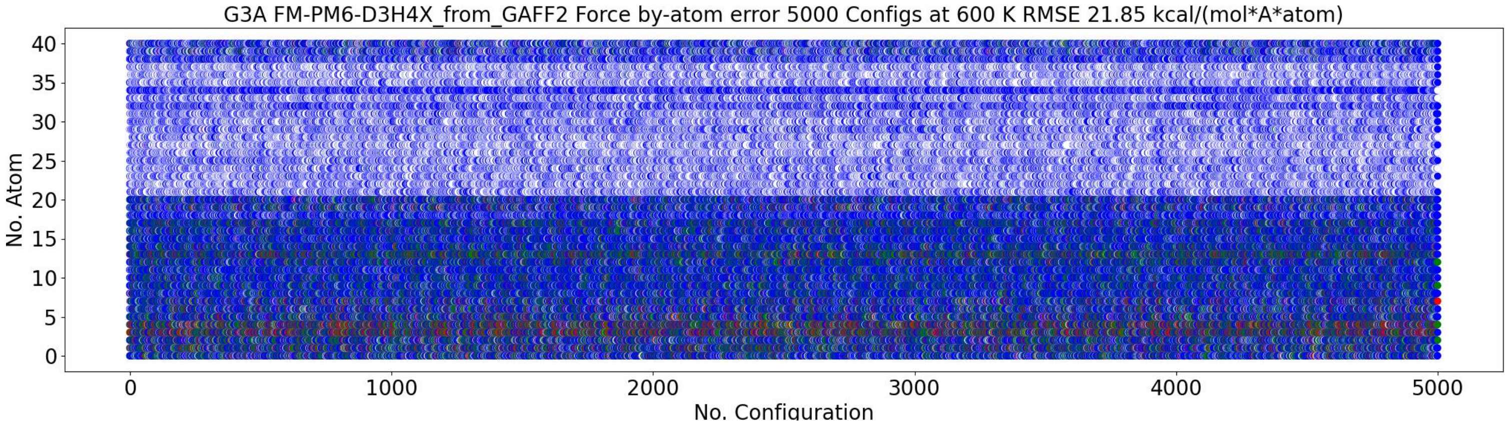

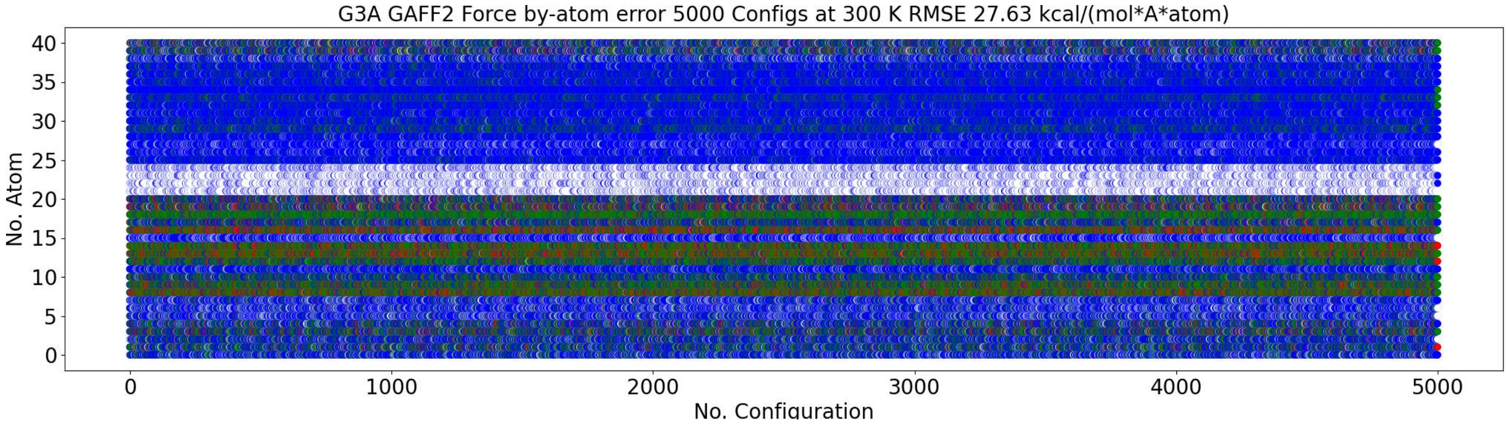

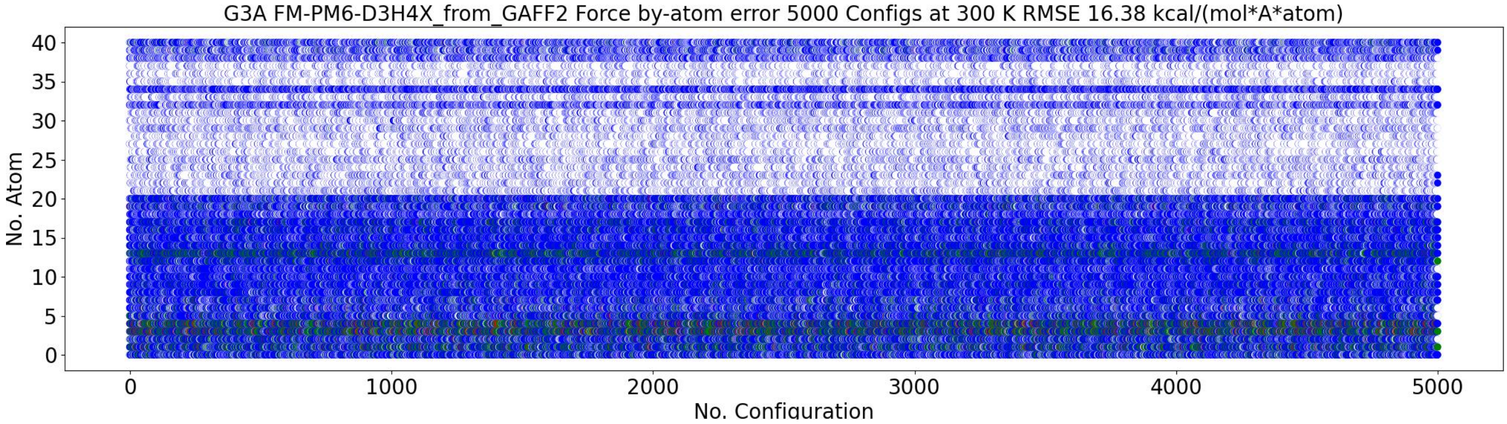

>50 kcal/(mol·Å) >30 kcal/(mol·Å) >10 kcal/(mol·Å)

G4A GAFF2 Force by-atom error 5000 Configs at 600 K RMSE 32.50 kcal/(mol*A*atom)

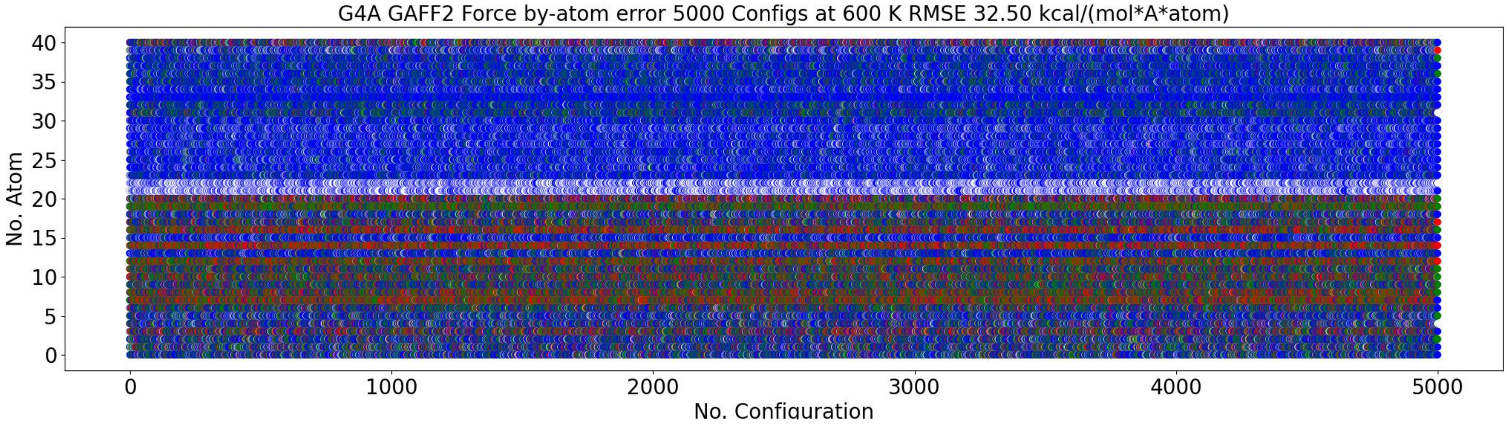

G4A FM-PM6-D3H4X_from_GAFF2 Force by-atom error 5000 Configs at 600 K RMSE 20.97 kcal/(mol*A*atom)

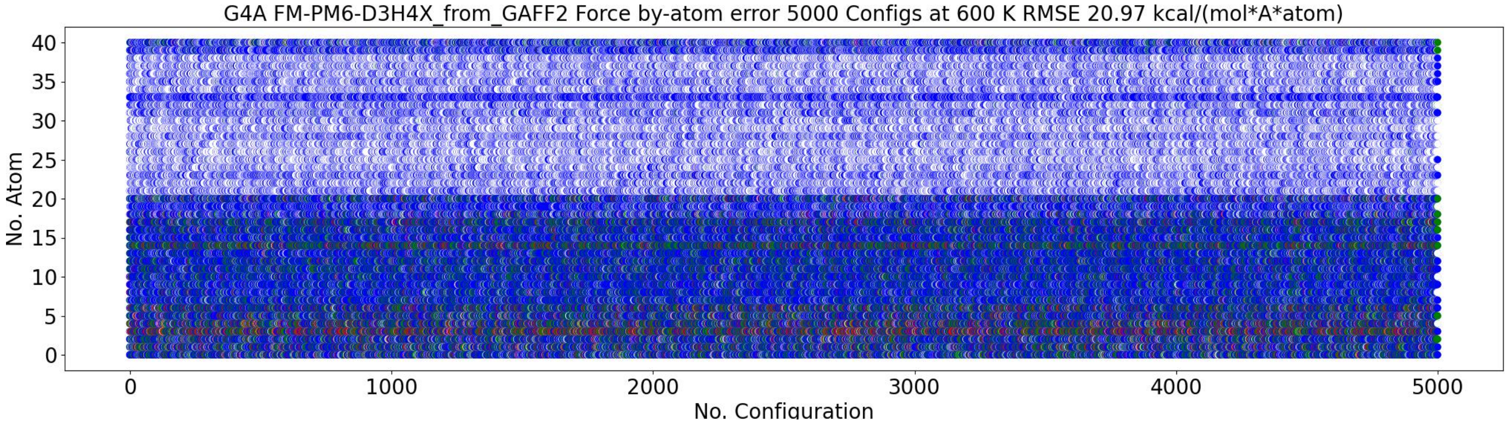

G4A GAFF2 Force by-atom error 5000 Configs at 300 K RMSE 28.11 kcal/(mol*A*atom)

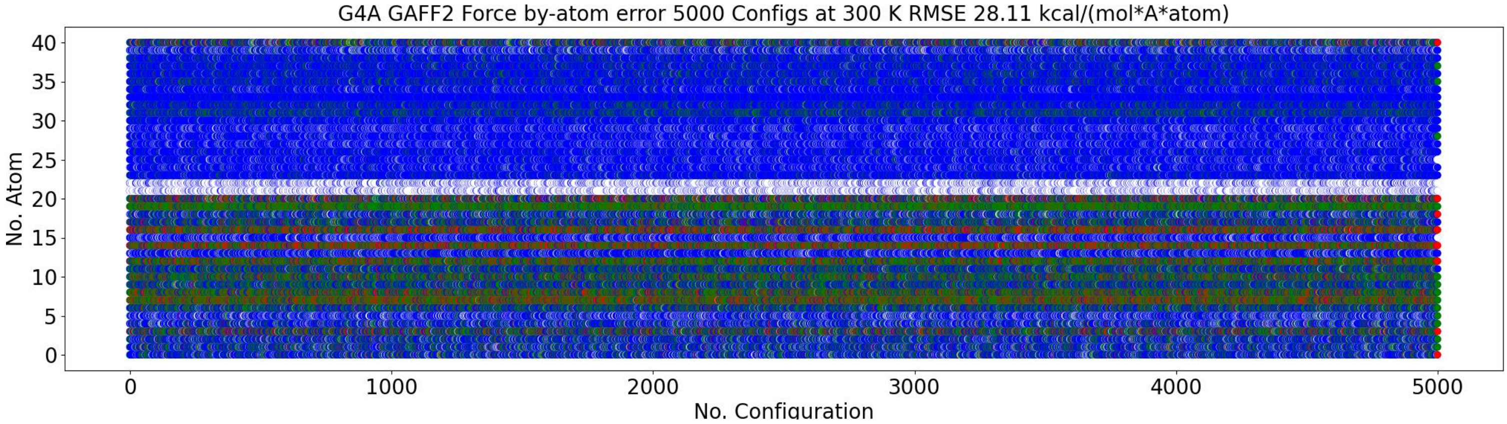

G4A FM-PM6-D3H4X_from_GAFF2 Force by-atom error 5000 Configs at 300 K RMSE 15.65 kcal/(mol*A*atom)

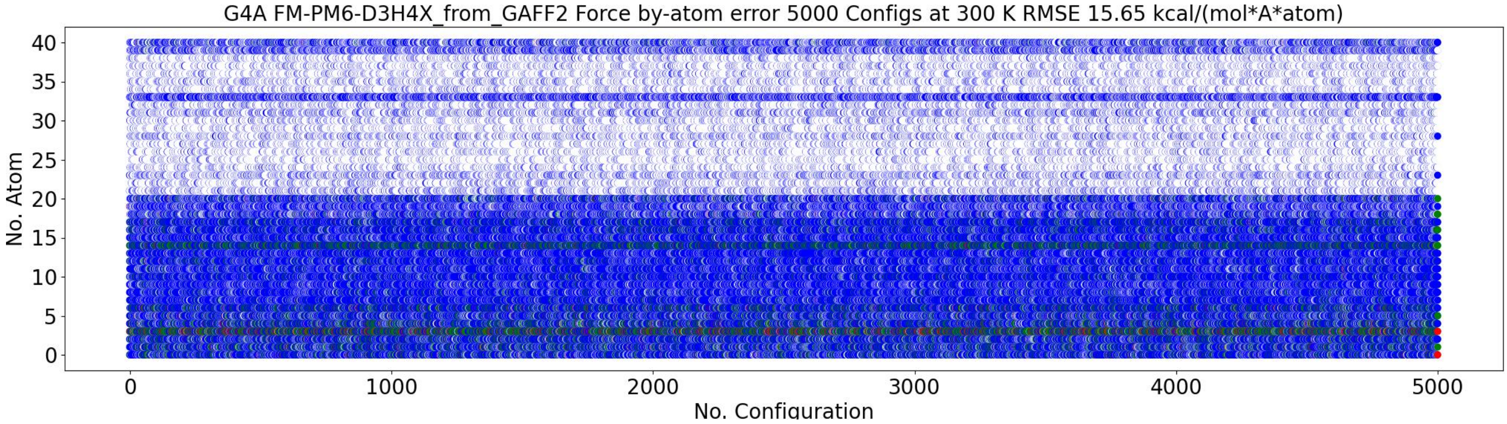

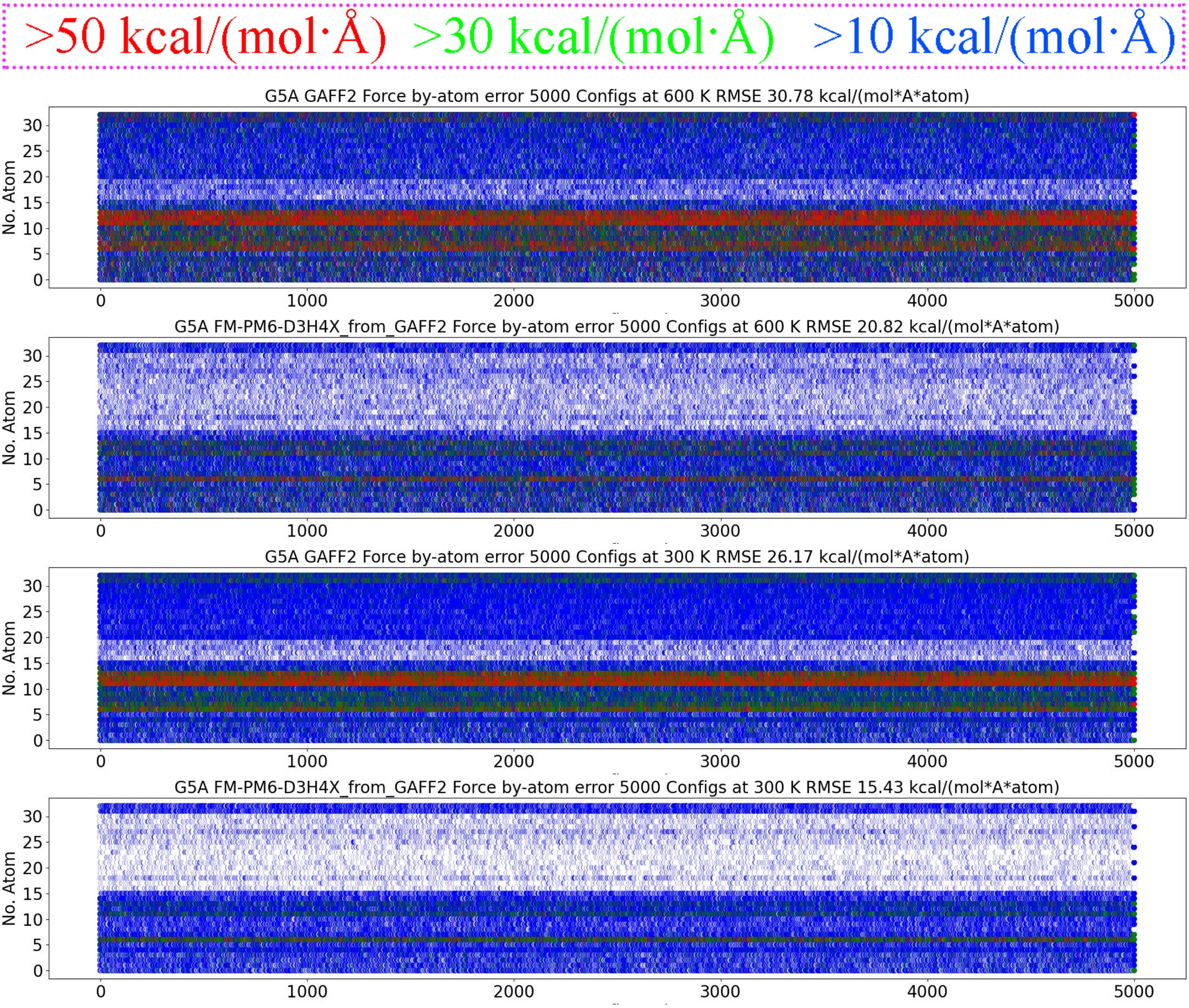
>50 kcal/(mol·Å)
>30 kcal/(mol·Å)
>10 kcal/(mol·Å)
G5A GAFF2 Force by-atom error 5000 Configs at 600 K RMSE 30.78 kcal/(mol*A*atom)
G5A FM-PM6-D3H4X_from_GAFF2 Force by-atom error 5000 Configs at 600 K RMSE 20.82 kcal/(mol*A*atom)
G5A GAFF2 Force by-atom error 5000 Configs at 300 K RMSE 26.17 kcal/(mol*A*atom)
G5A FM-PM6-D3H4X_from_GAFF2 Force by-atom error 5000 Configs at 300 K RMSE 15.43 kcal/(mol*A*atom)
No. Atom
0
5
10
15
20
25
30
1000
2000
3000
4000
5000

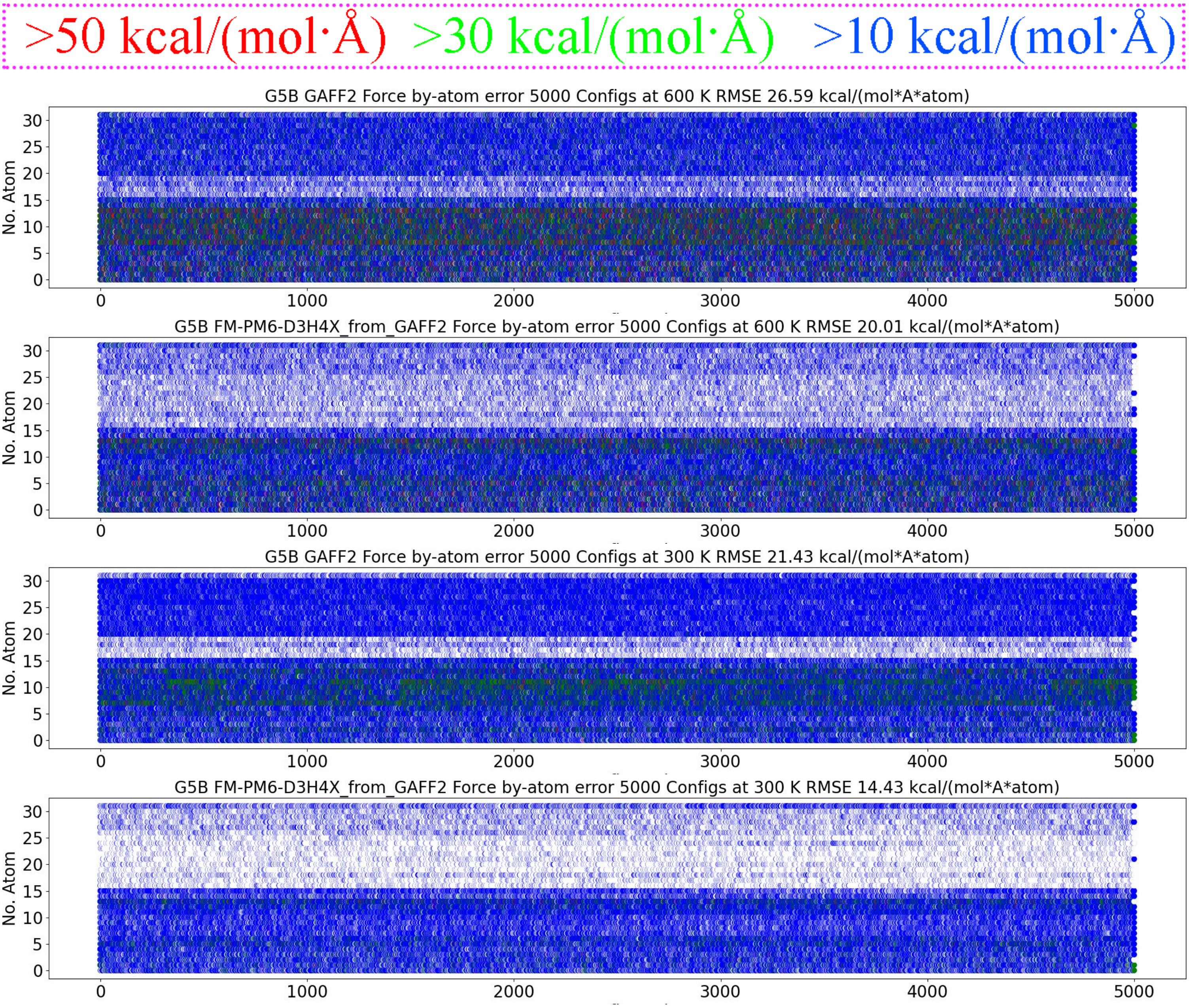
>50 kcal/(mol·Å) >30 kcal/(mol·Å) >10 kcal/(mol·Å)
G5B GAFF2 Force by-atom error 5000 Configs at 600 K RMSE 26.59 kcal/(mol*A*atom)
G5B FM-PM6-D3H4X_from_GAFF2 Force by-atom error 5000 Configs at 600 K RMSE 20.01 kcal/(mol*A*atom)
G5B GAFF2 Force by-atom error 5000 Configs at 300 K RMSE 21.43 kcal/(mol*A*atom)
G5B FM-PM6-D3H4X_from_GAFF2 Force by-atom error 5000 Configs at 300 K RMSE 14.43 kcal/(mol*A*atom)
No. Atom

>50 kcal/(mol·Å) >30 kcal/(mol·Å) >10 kcal/(mol·Å)

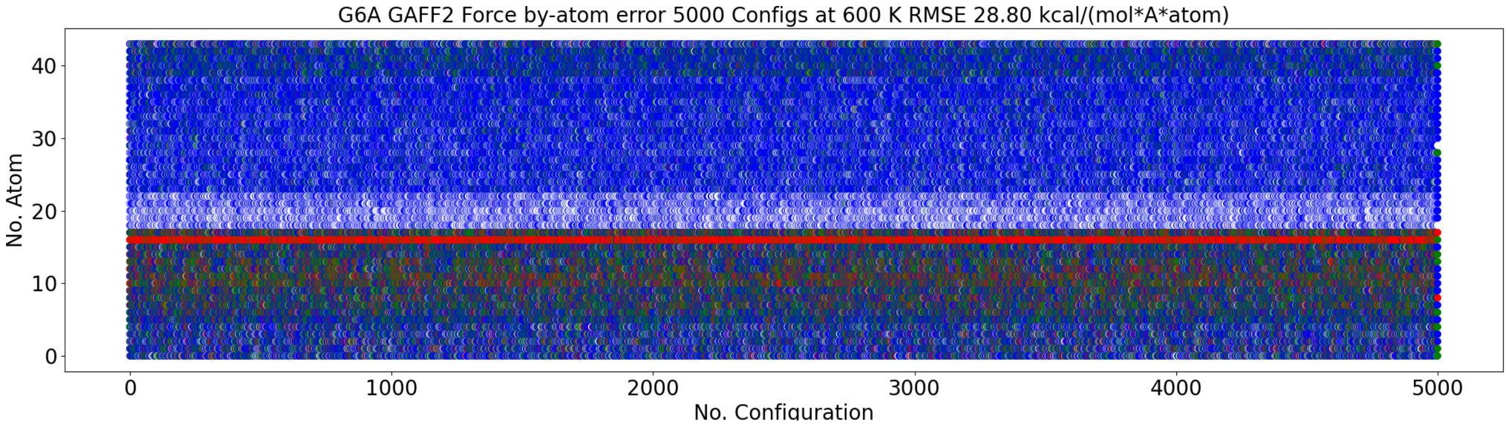

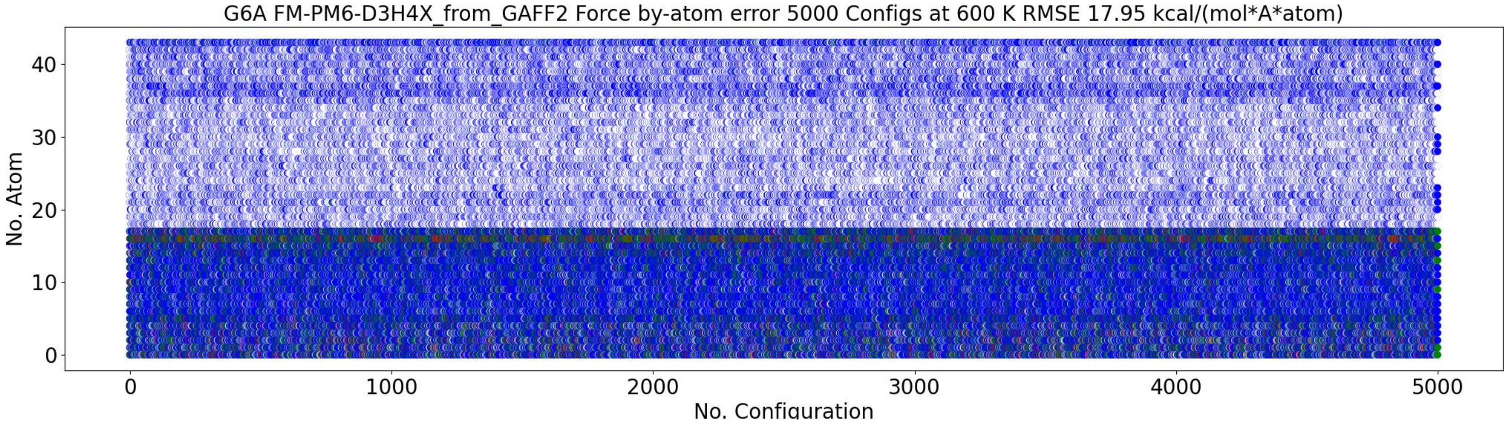

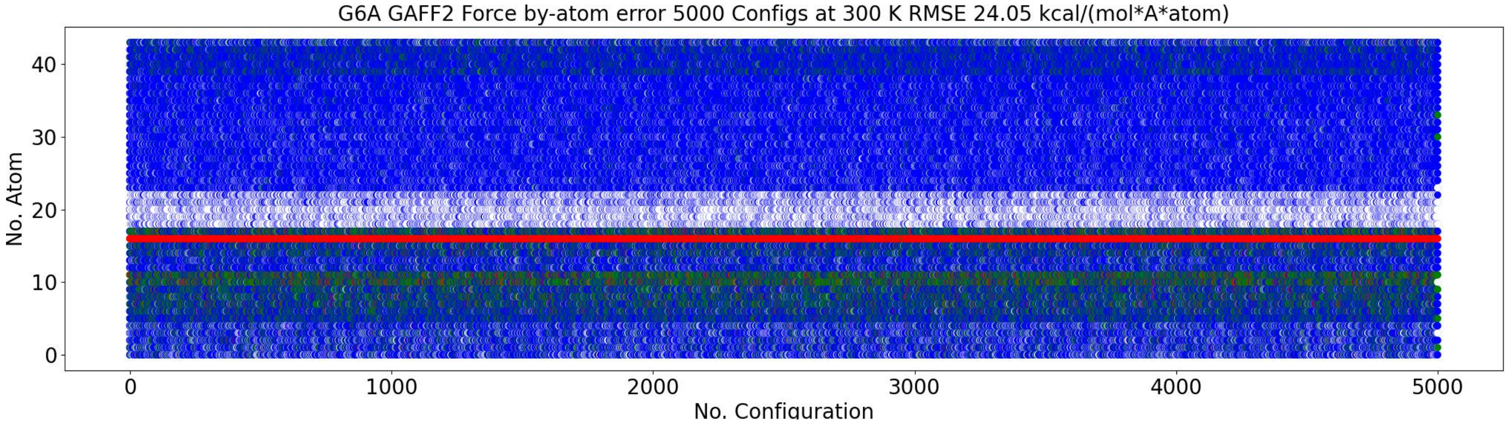

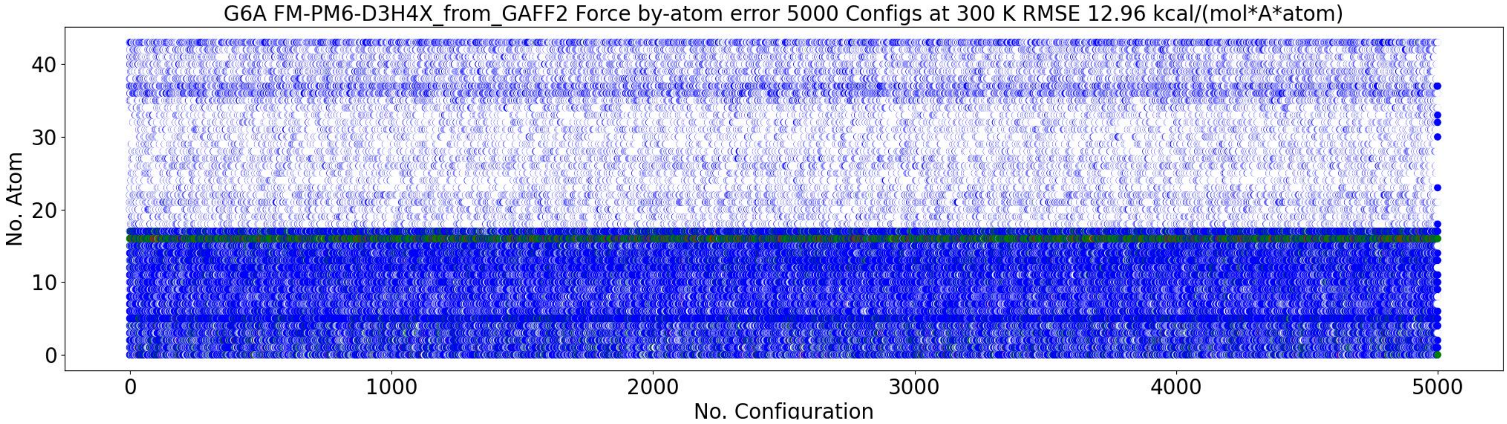

>50 kcal/(mol·Å) >30 kcal/(mol·Å) >10 kcal/(mol·Å)

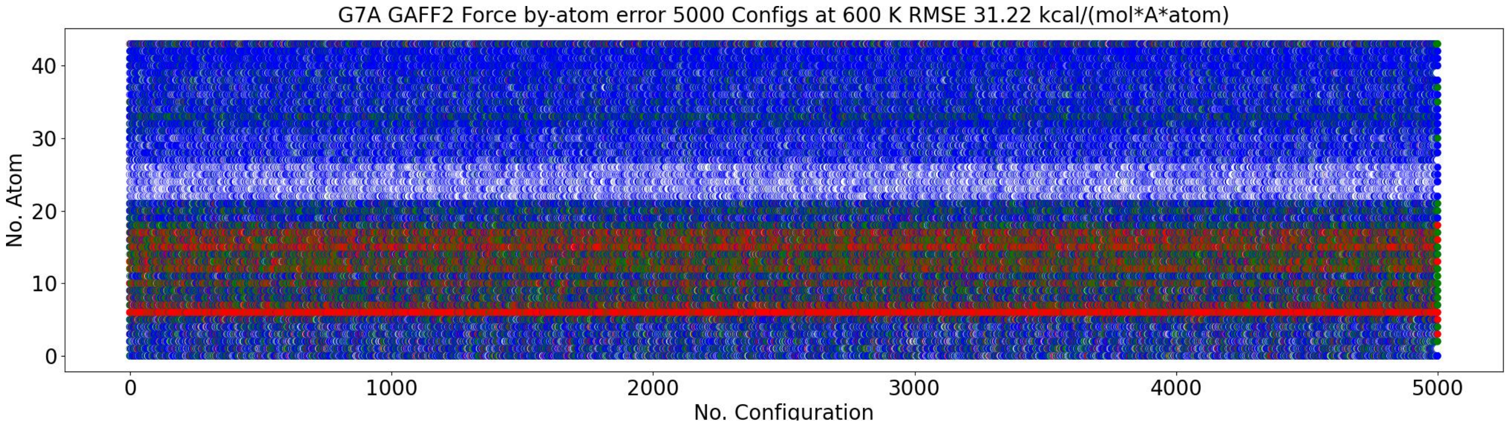

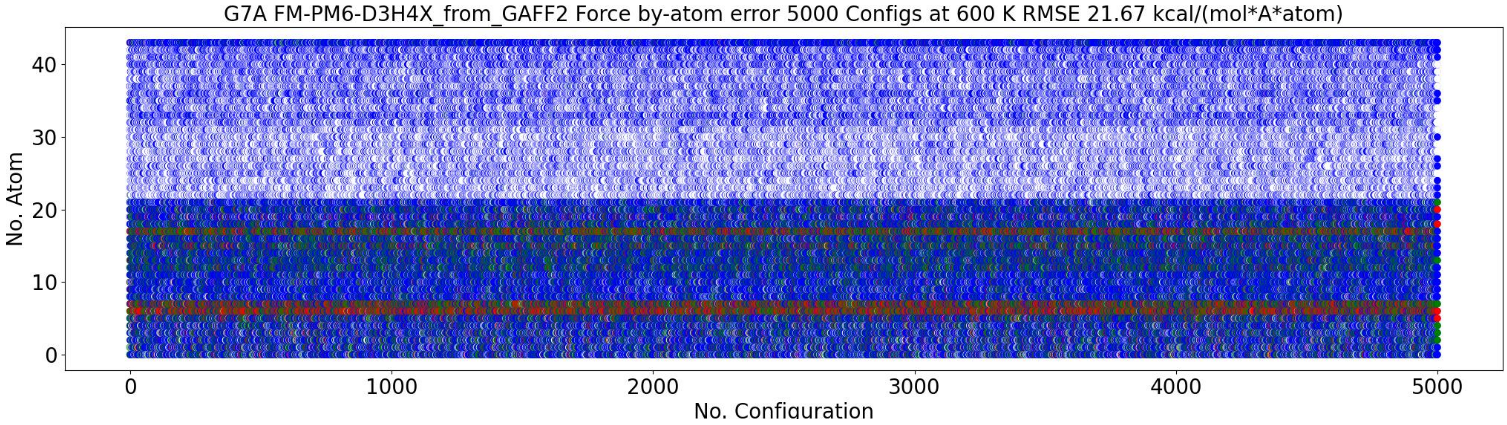

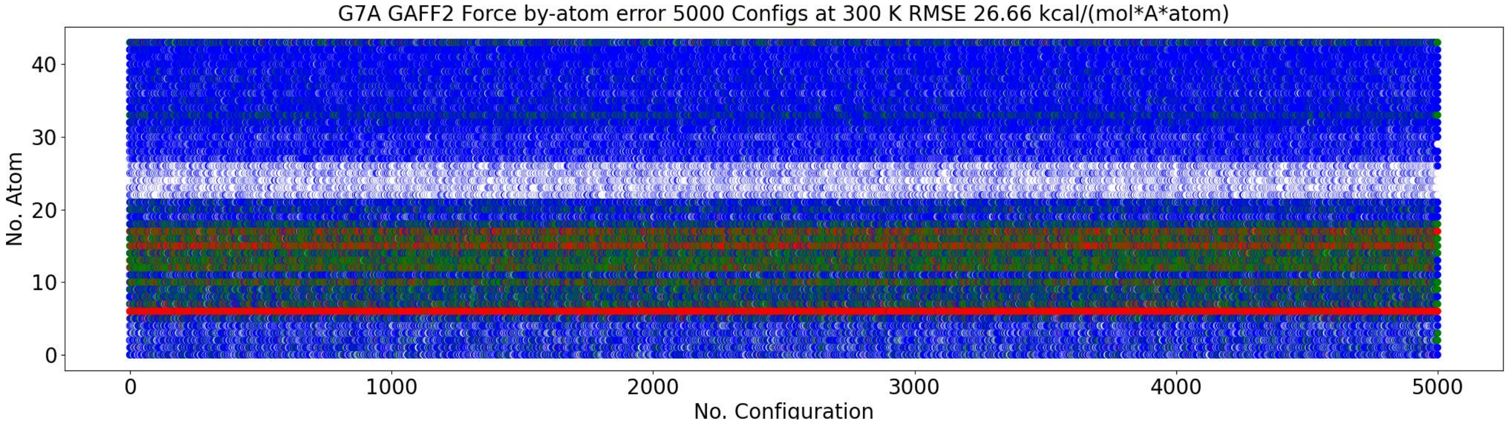

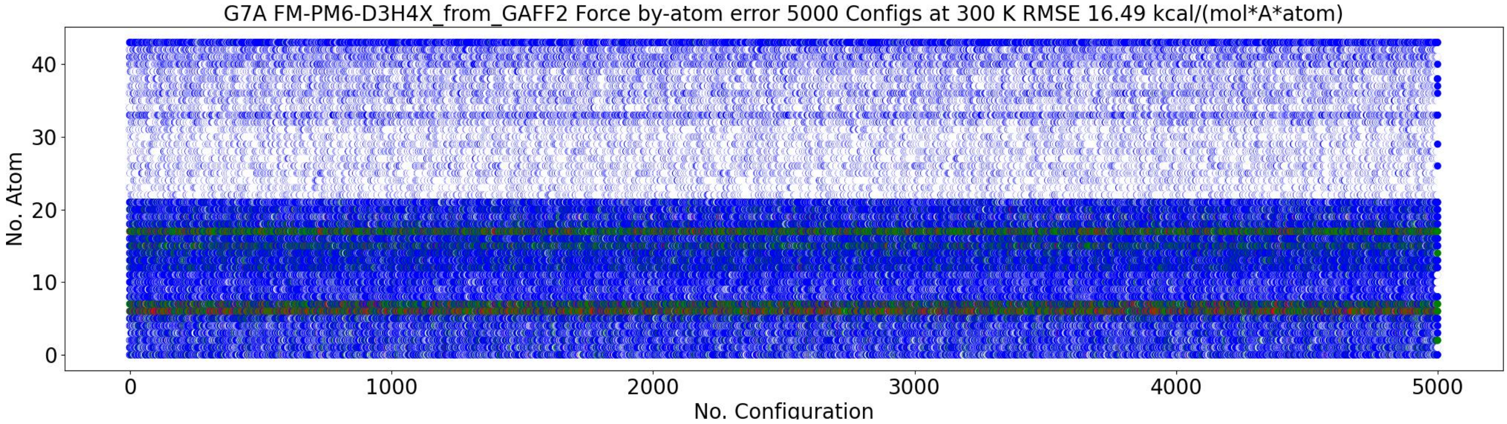

Fig. S3. The correlations between the MM and ab initio QM (BLYP-D4) energetics calculated from 10 ns trajectories generated at 300 K in vacuo for the host and 7 guest molecules. The sampling interval is 10 ps and there are 1000 independent configurations in total. The RMSE and MAE of the original parameter set and the newly fitted force-matching set are also presented. Note that the protonated and deprotonated forms of G5 Ketamine are fitted separately.

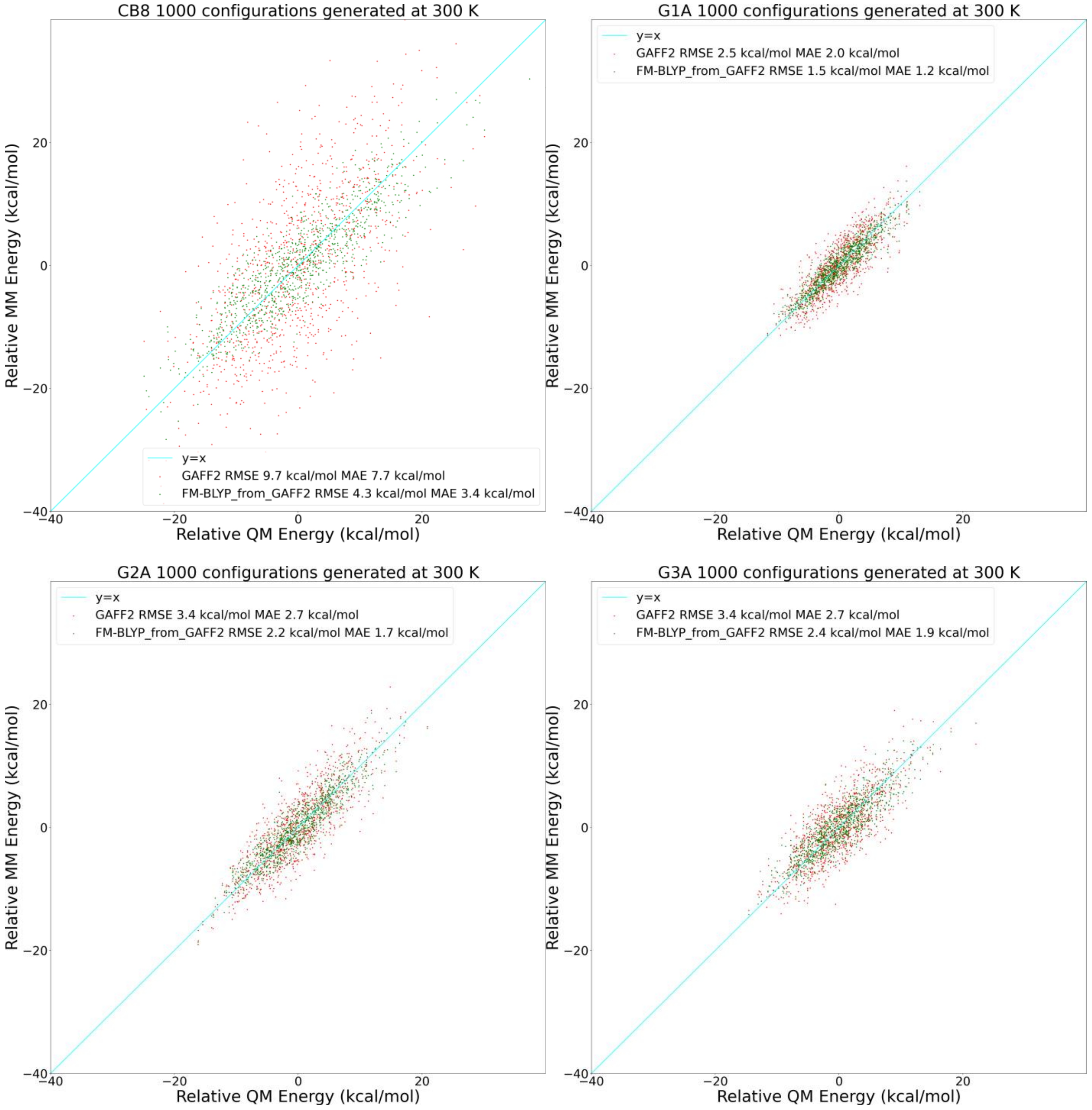

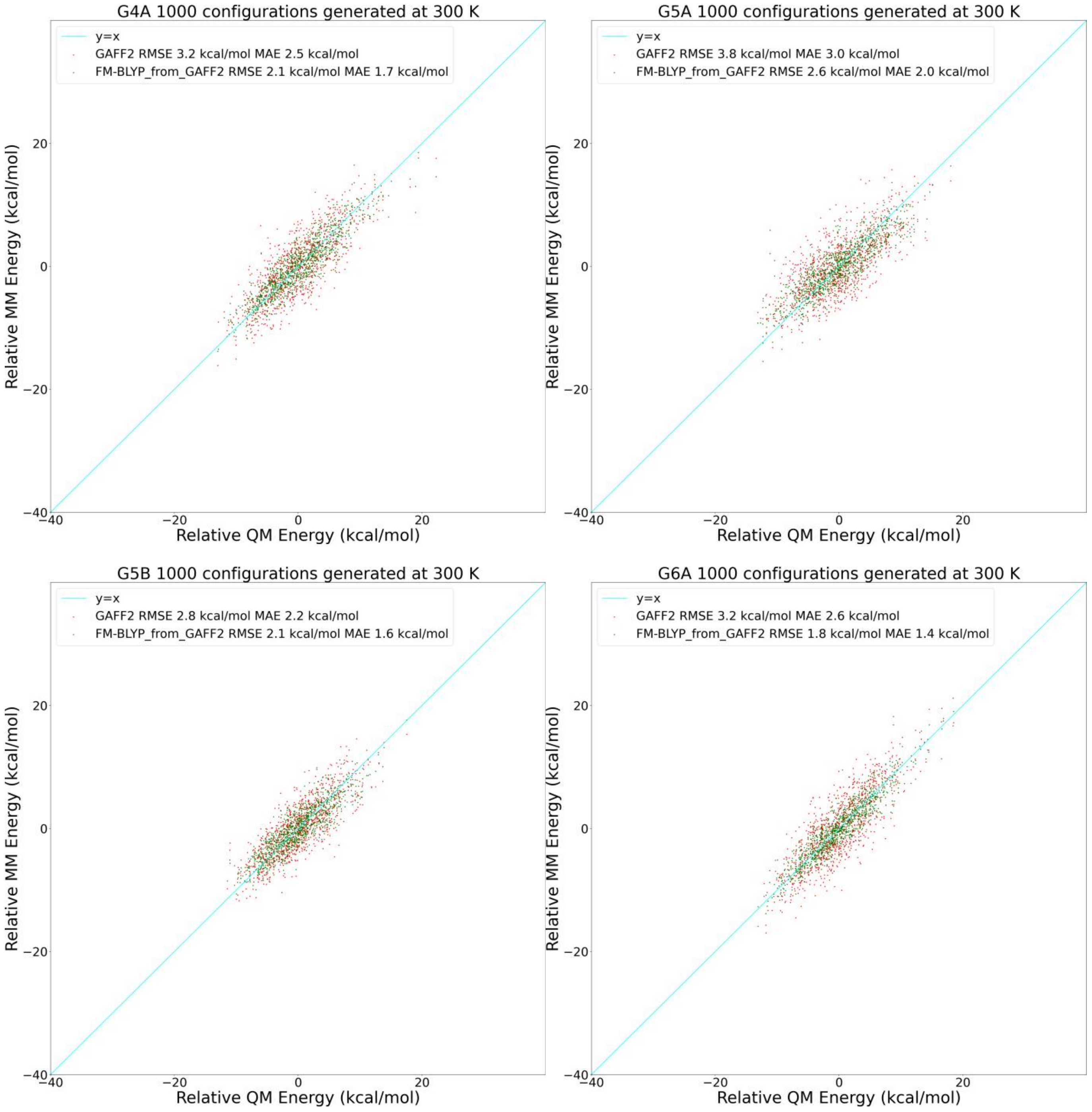
G4A 1000 configurations generated at 300 K
y=x
GAFF2 RMSE 3.2 kcal/mol MAE 2.5 kcal/mol
FM-BLYP_from_GAFF2 RMSE 2.1 kcal/mol MAE 1.7 kcal/mol
G5A 1000 configurations generated at 300 K
y=x
GAFF2 RMSE 3.8 kcal/mol MAE 3.0 kcal/mol
FM-BLYP_from_GAFF2 RMSE 2.6 kcal/mol MAE 2.0 kcal/mol
G5B 1000 configurations generated at 300 K
y=x
GAFF2 RMSE 2.8 kcal/mol MAE 2.2 kcal/mol
FM-BLYP_from_GAFF2 RMSE 2.1 kcal/mol MAE 1.6 kcal/mol
G6A 1000 configurations generated at 300 K
y=x
GAFF2 RMSE 3.2 kcal/mol MAE 2.6 kcal/mol
FM-BLYP_from_GAFF2 RMSE 1.8 kcal/mol MAE 1.4 kcal/mol
Relative MM Energy (kcal/mol)
Relative QM Energy (kcal/mol)
20
0
−20
−40

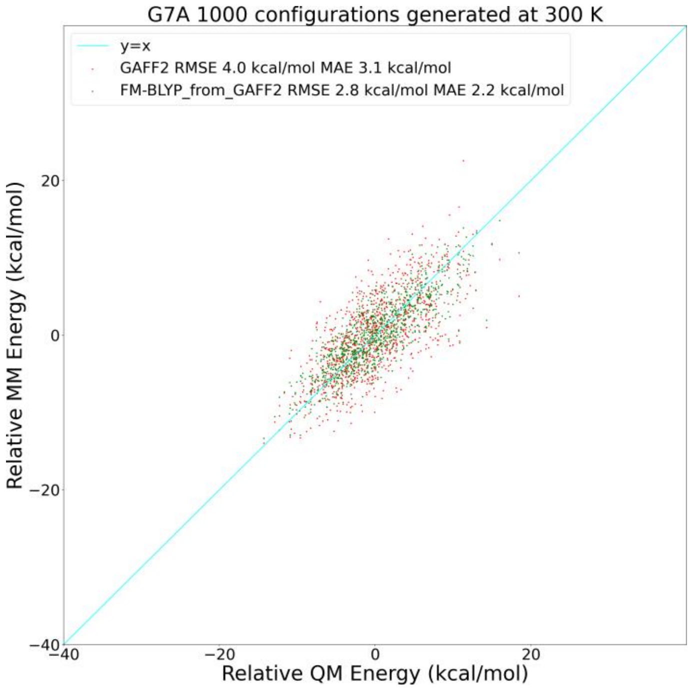
G7A 1000 configurations generated at 300 K
y=x
GAFF2 RMSE 4.0 kcal/mol MAE 3.1 kcal/mol
FM-BLYP_from_GAFF2 RMSE 2.8 kcal/mol MAE 2.2 kcal/mol
Relative MM Energy (kcal/mol)
Relative QM Energy (kcal/mol)
20
0
−20
−40
−40
−20
0
20

Fig. S4. The time series of the errors of atomic forces ($\|\Delta \mathbf{F}_i\|_2$ for the *i*th atom) under the original GAFF2 and the refitted FM-BLYP_from_GAFF2 parameter set calculated from 10 ns trajectories generated at 300 K in vacuo for the host and guest molecules. The protonated and deprotonated forms of G5 Ketamine are parameterized and thus tested separately. The sampling interval is 10 ps and there are 1000 independent configurations in total. Red dots for the force errors larger than 50 kcal/(mol·Å), green for force errors larger than 30 kcal/(mol·Å), blue for errors larger than 10 kcal/(mol·Å), and white for the other small-error points. The overall RMSEs of atomic forces of each molecule in kcal/(mol·Å·atom) under the original GAFF2 and the newly obtained FM-BLYP parameter sets are also given.

>50 kcal/(mol·Å) >30 kcal/(mol·Å) >10 kcal/(mol·Å)

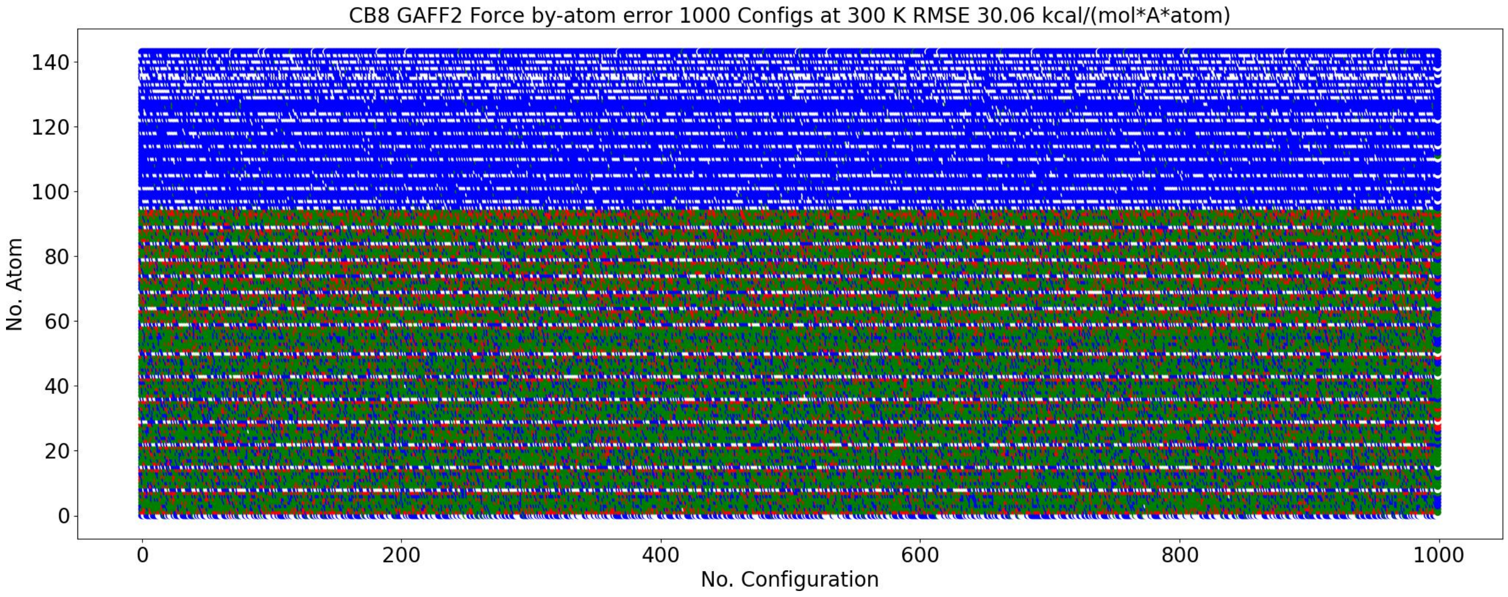

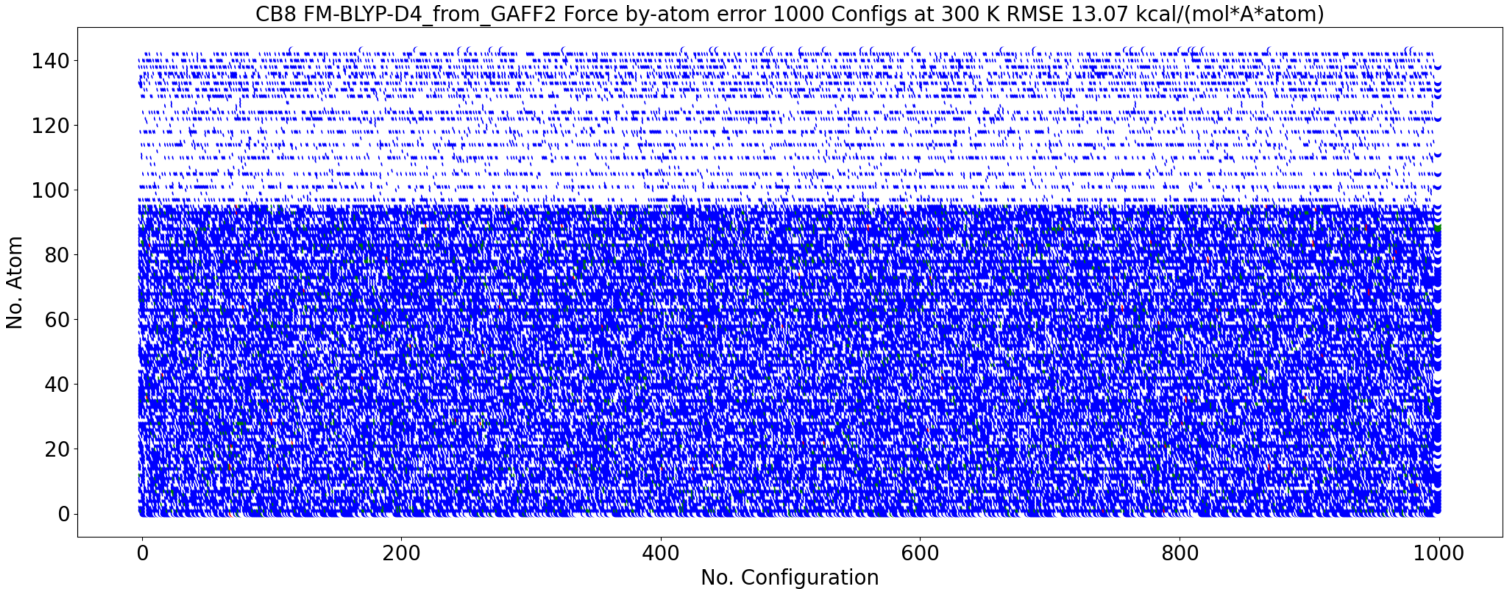

>50 kcal/(mol·Å) >30 kcal/(mol·Å) >10 kcal/(mol·Å)

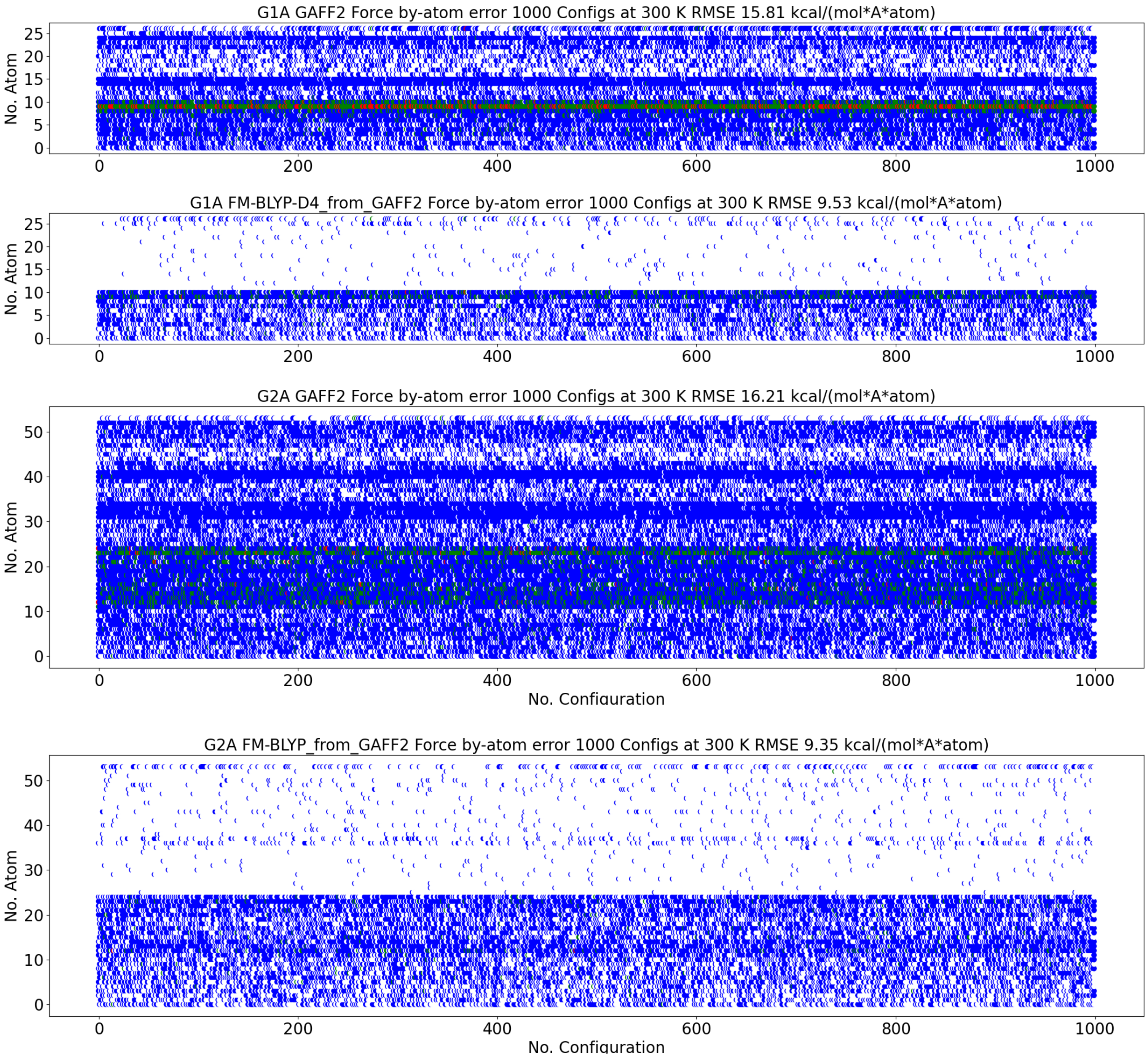

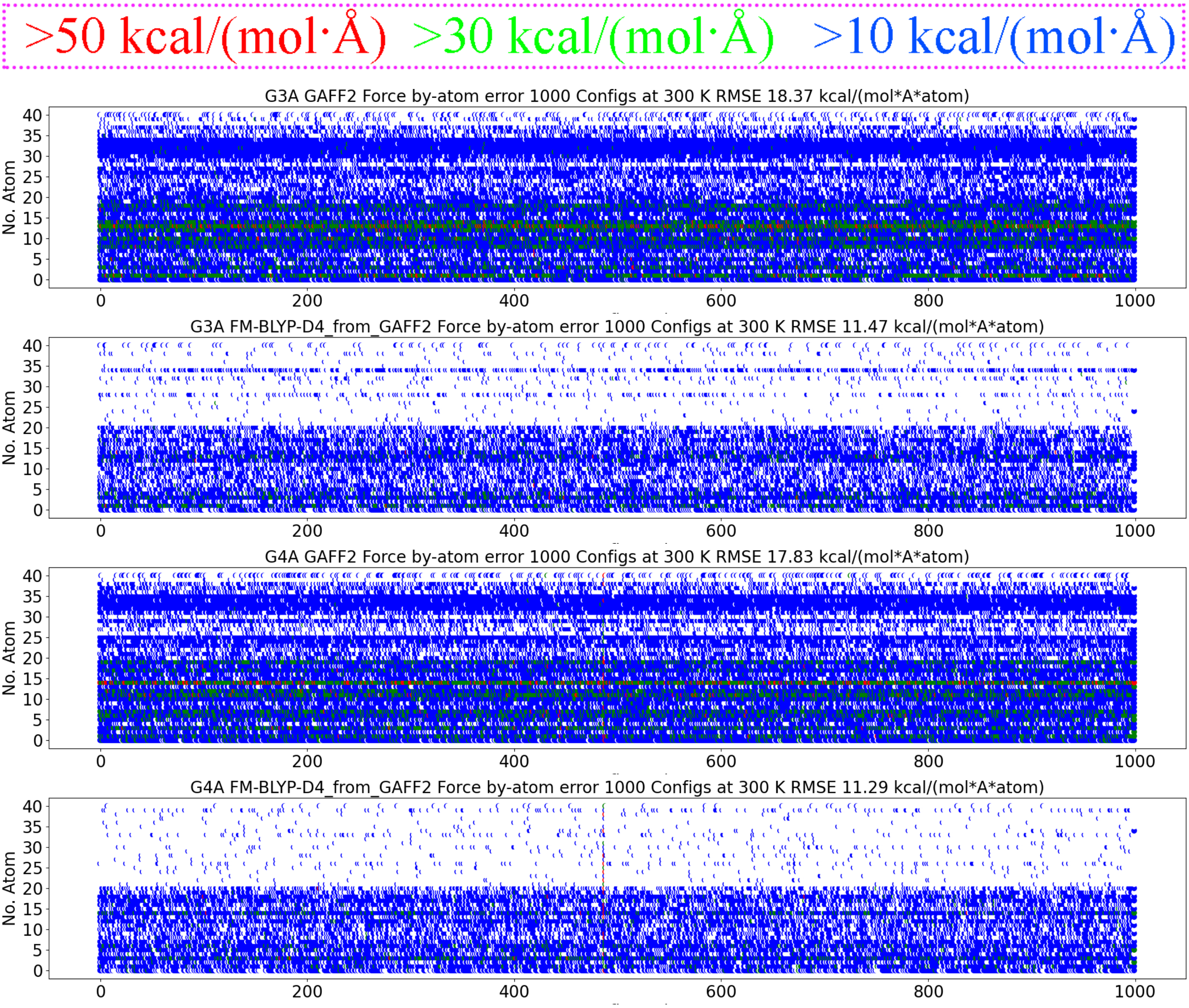
>50 kcal/(mol·Å)
>30 kcal/(mol·Å)
>10 kcal/(mol·Å)
G3A GAFF2 Force by-atom error 1000 Configs at 300 K RMSE 18.37 kcal/(mol*A*atom)
G3A FM-BLYP-D4_from_GAFF2 Force by-atom error 1000 Configs at 300 K RMSE 11.47 kcal/(mol*A*atom)
G4A GAFF2 Force by-atom error 1000 Configs at 300 K RMSE 17.83 kcal/(mol*A*atom)
G4A FM-BLYP-D4_from_GAFF2 Force by-atom error 1000 Configs at 300 K RMSE 11.29 kcal/(mol*A*atom)
No. Atom

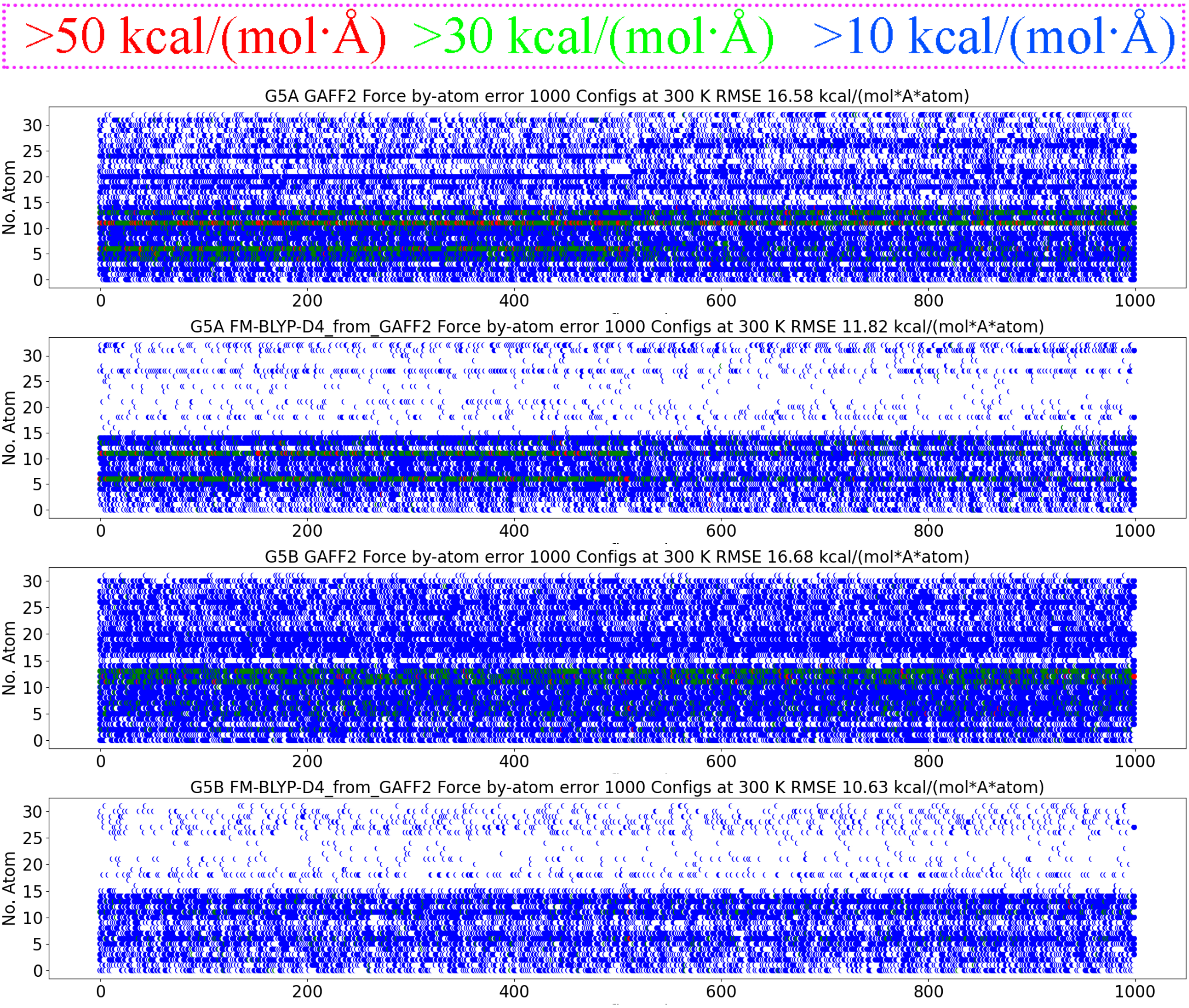
>50 kcal/(mol·Å)
>30 kcal/(mol·Å)
>10 kcal/(mol·Å)
G5A GAFF2 Force by-atom error 1000 Configs at 300 K RMSE 16.58 kcal/(mol*A*atom)
G5A FM-BLYP-D4_from_GAFF2 Force by-atom error 1000 Configs at 300 K RMSE 11.82 kcal/(mol*A*atom)
G5B GAFF2 Force by-atom error 1000 Configs at 300 K RMSE 16.68 kcal/(mol*A*atom)
G5B FM-BLYP-D4_from_GAFF2 Force by-atom error 1000 Configs at 300 K RMSE 10.63 kcal/(mol*A*atom)
No. Atom
0
5
10
15
20
25
30
0
200
400
600
800
1000

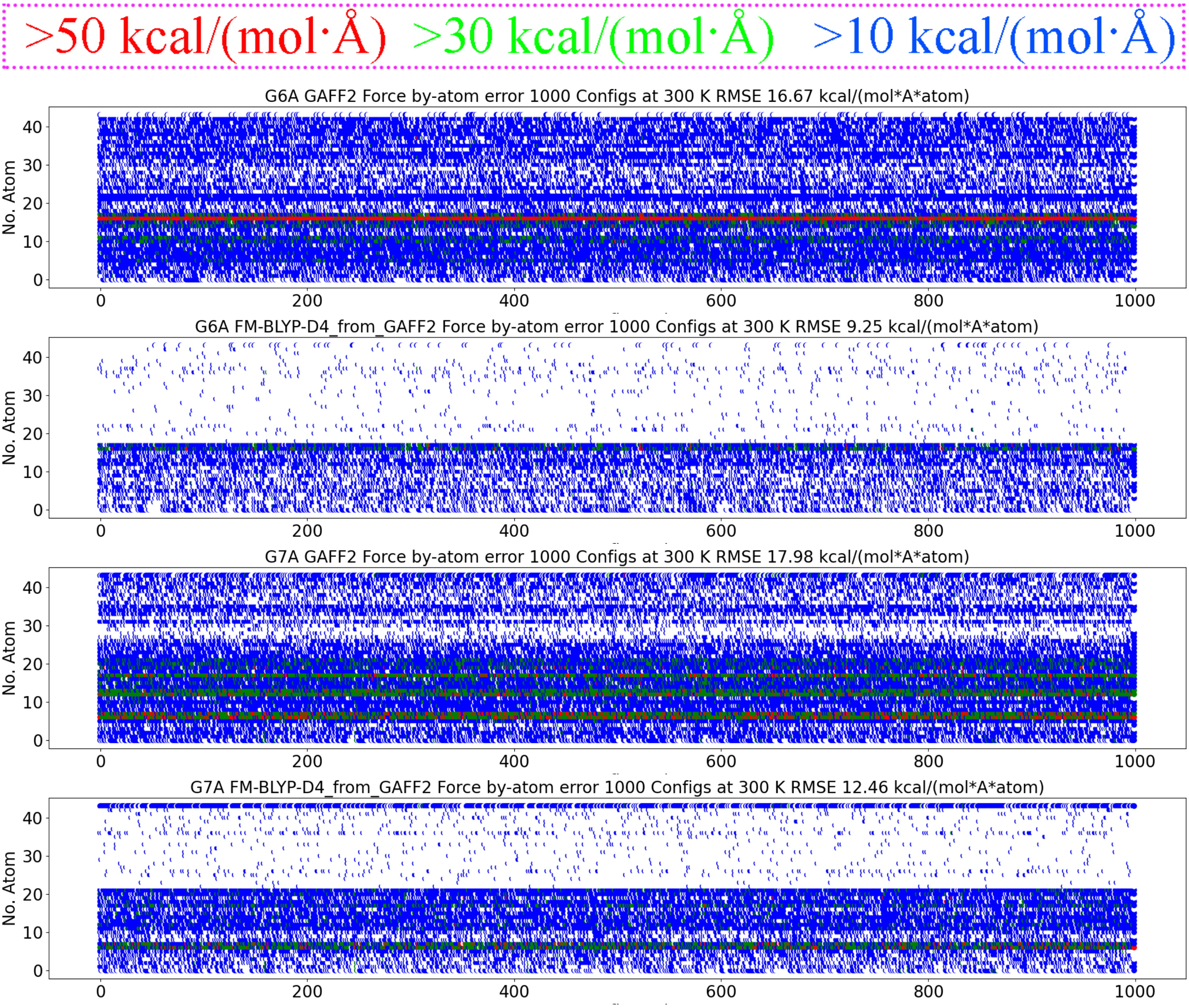
>50 kcal/(mol·Å)
>30 kcal/(mol·Å)
>10 kcal/(mol·Å)
G6A GAFF2 Force by-atom error 1000 Configs at 300 K RMSE 16.67 kcal/(mol*A*atom)
G6A FM-BLYP-D4_from_GAFF2 Force by-atom error 1000 Configs at 300 K RMSE 9.25 kcal/(mol*A*atom)
G7A GAFF2 Force by-atom error 1000 Configs at 300 K RMSE 17.98 kcal/(mol*A*atom)
G7A FM-BLYP-D4_from_GAFF2 Force by-atom error 1000 Configs at 300 K RMSE 12.46 kcal/(mol*A*atom)
No. Atom
0
10
20
30
40
0
200
400
600
800
1000

Fig. S5. Dihedral terms defined in GAFF derivatives describing the intra-molecular conformational preference of the host CB8.

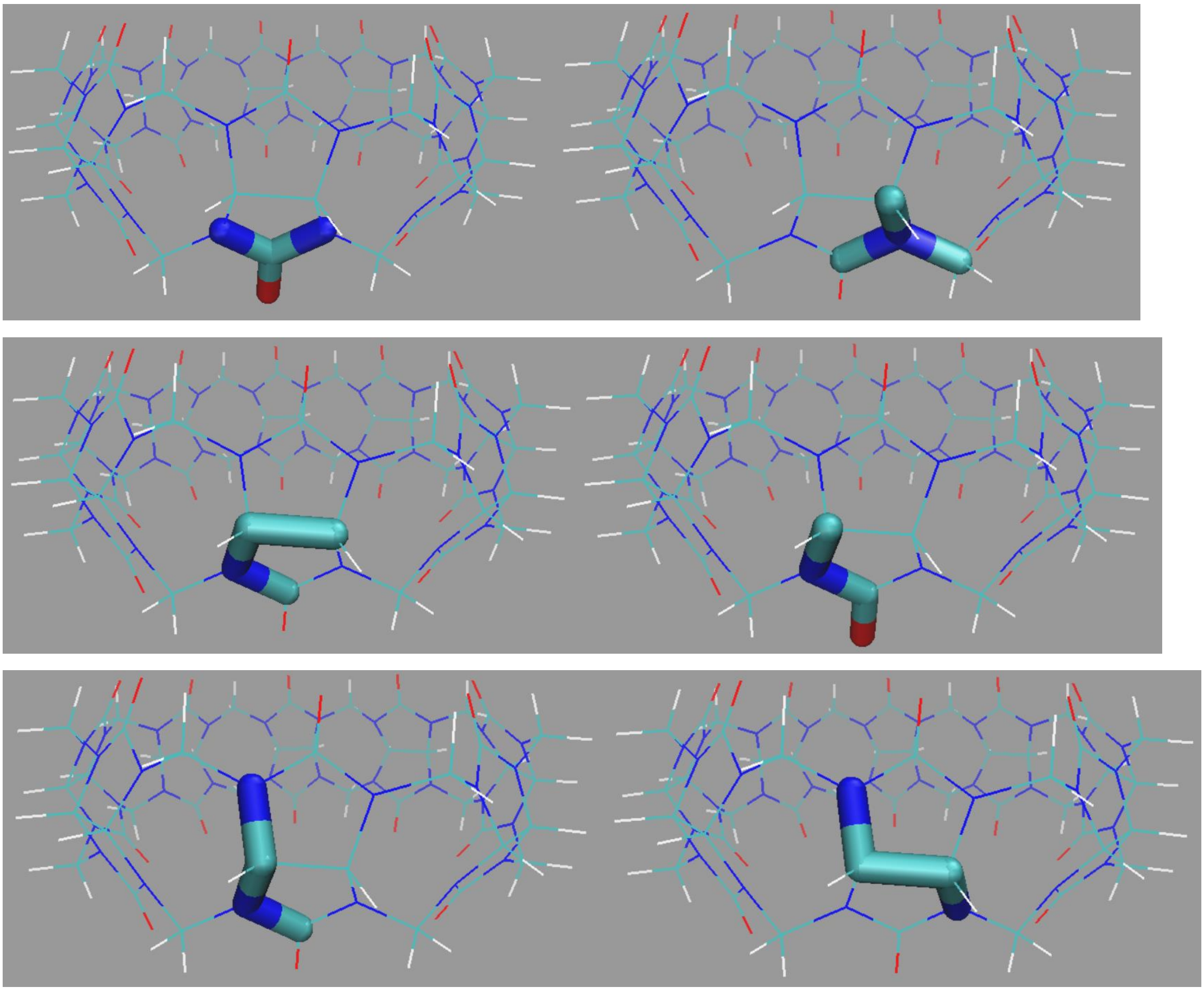

Fig. S6. The number of contacts between all atoms of the host CB8 and the guests and the by-host-atom decomposition during metadynamics simulations under the GAFF2 force field. The y-axis represents the serial number of host atom. Among the 144 atoms of the host CB8, the first 96 atoms are heavy atoms, and the other 48 atoms are hydrogen atoms. All atoms of the host and the guest are included in the calculation. Red dots denote contacts larger than 10, green dots represent contact number between 5 and 10, blue ones are those larger than 1, and the other are represented by white dots.

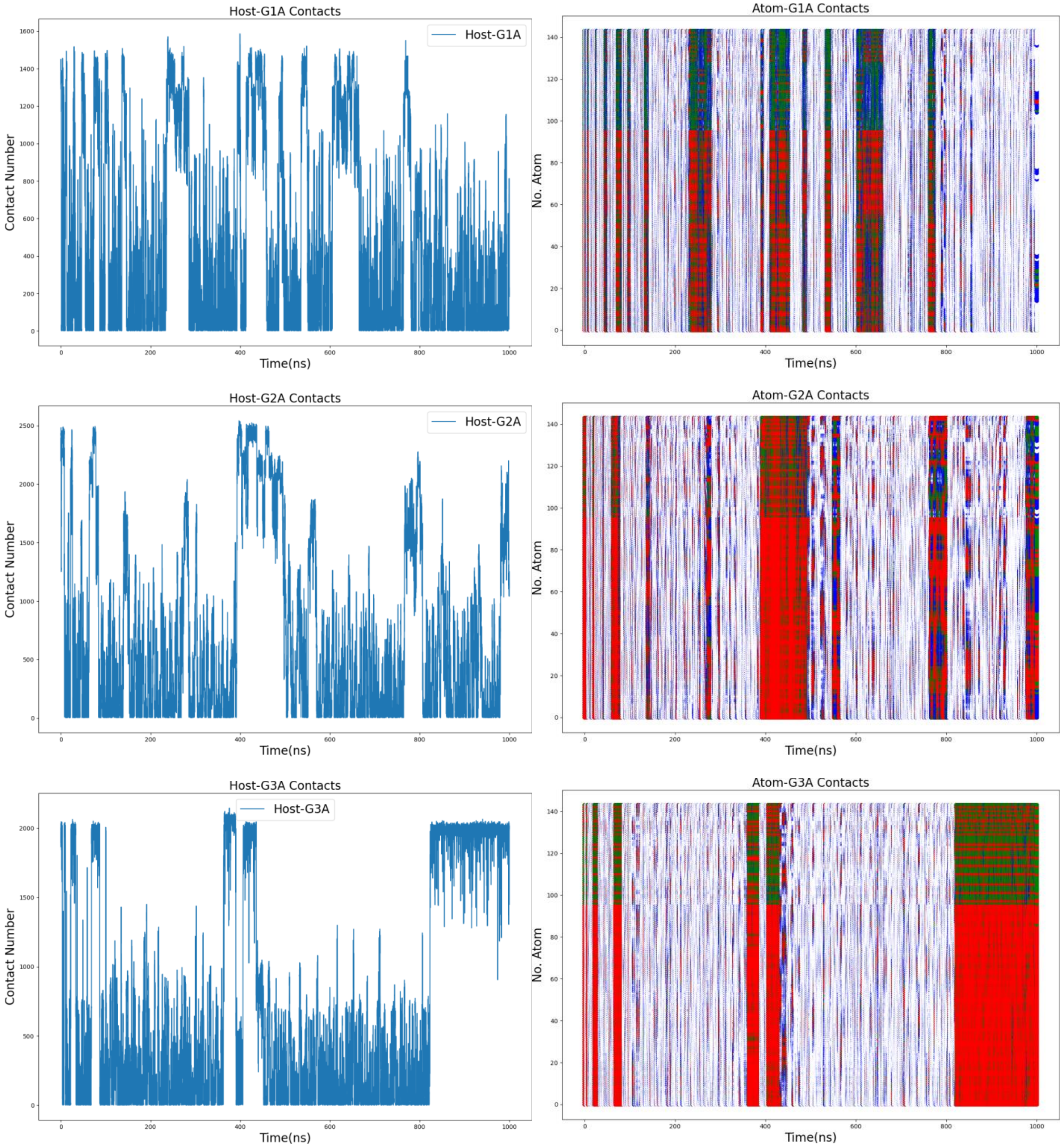

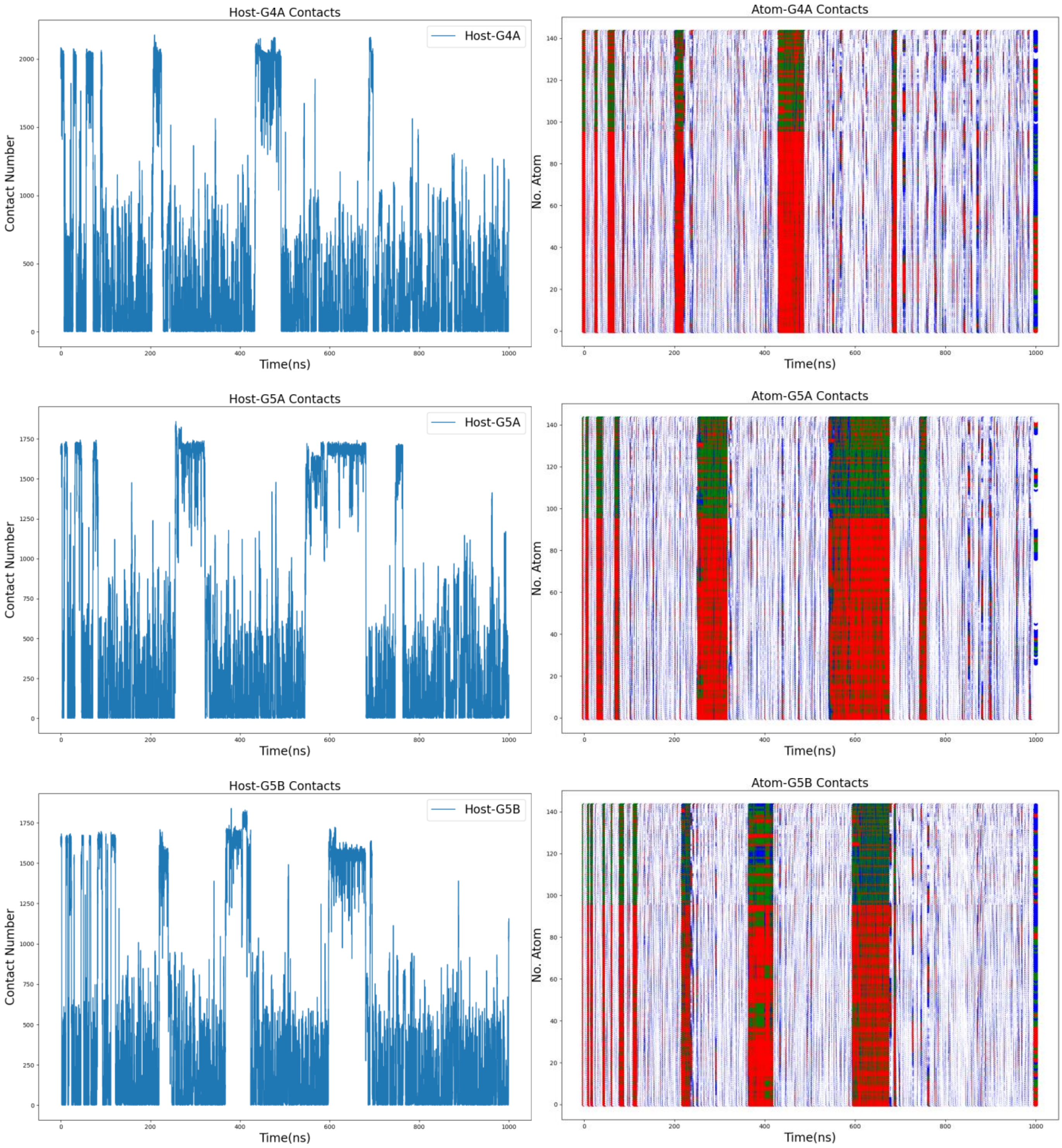
Host-G4A Contacts
Host-G4A
Contact Number
Time(ns)
Atom-G4A Contacts
No. Atom
Time(ns)
Host-G5A Contacts
Host-G5A
Contact Number
Time(ns)
Atom-G5A Contacts
No. Atom
Time(ns)
Host-G5B Contacts
Host-G5B
Contact Number
Time(ns)
Atom-G5B Contacts
No. Atom
Time(ns)

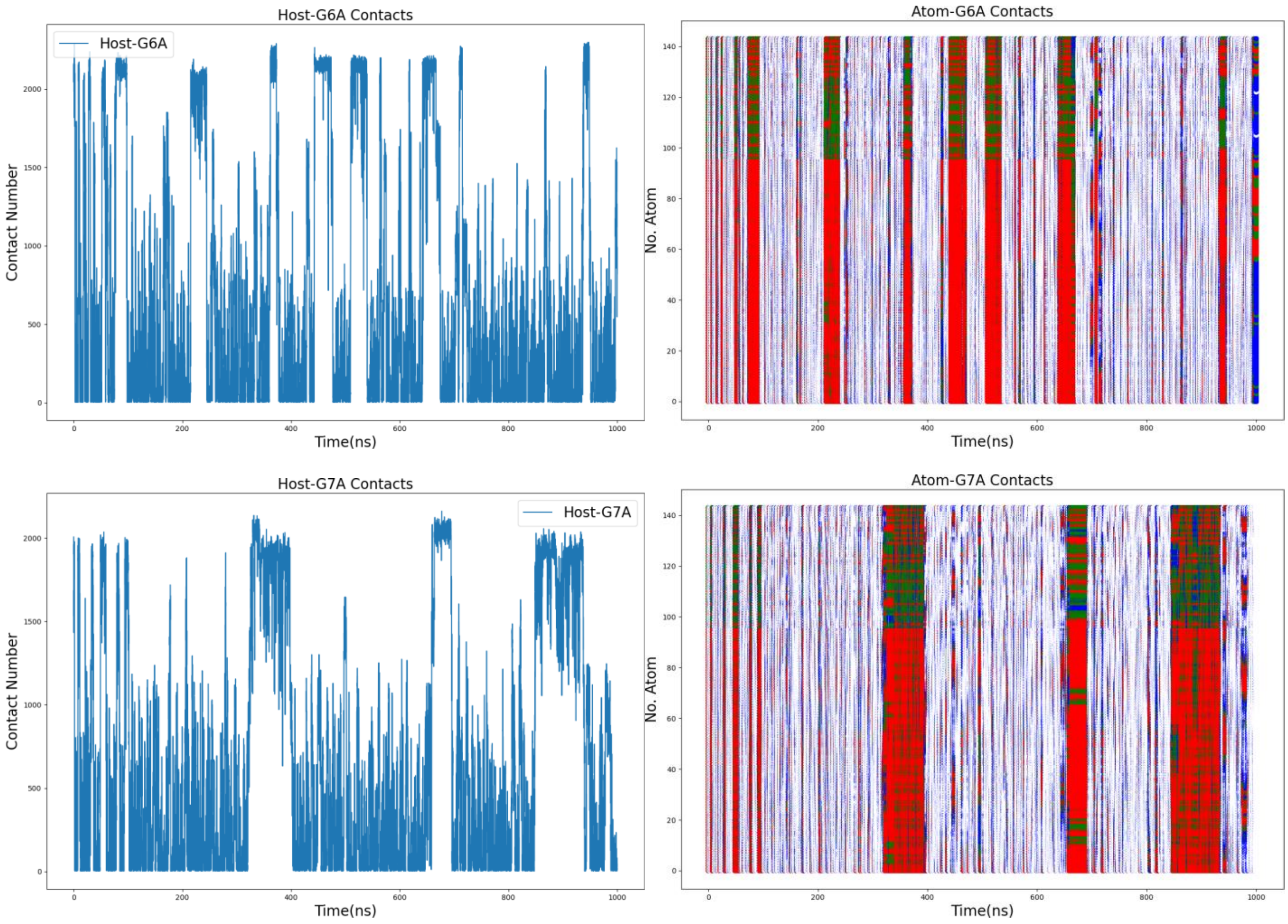
Host-G6A Contacts
Host-G6A
Contact Number
Time(ns)
Atom-G6A Contacts
No. Atom
Time(ns)
Host-G7A Contacts
Host-G7A
Contact Number
Time(ns)
Atom-G7A Contacts
No. Atom
Time(ns)

Fig. S7. The number of contacts between all atoms of the host CB8 and the guests and the by-host-atom decomposition during metadynamics simulations with the FM-PM6 parameter set. The y-axis represents the serial number of host atom. Among the 144 atoms of the host CB8, the first 96 atoms are heavy atoms, and the other 48 atoms are hydrogen atoms. All atoms of the host and the guest are included in the calculation. Red dots denote contacts larger than 10, green dots represent contact number between 5 and 10, blue ones are those larger than 1, and the other are represented by white dots.

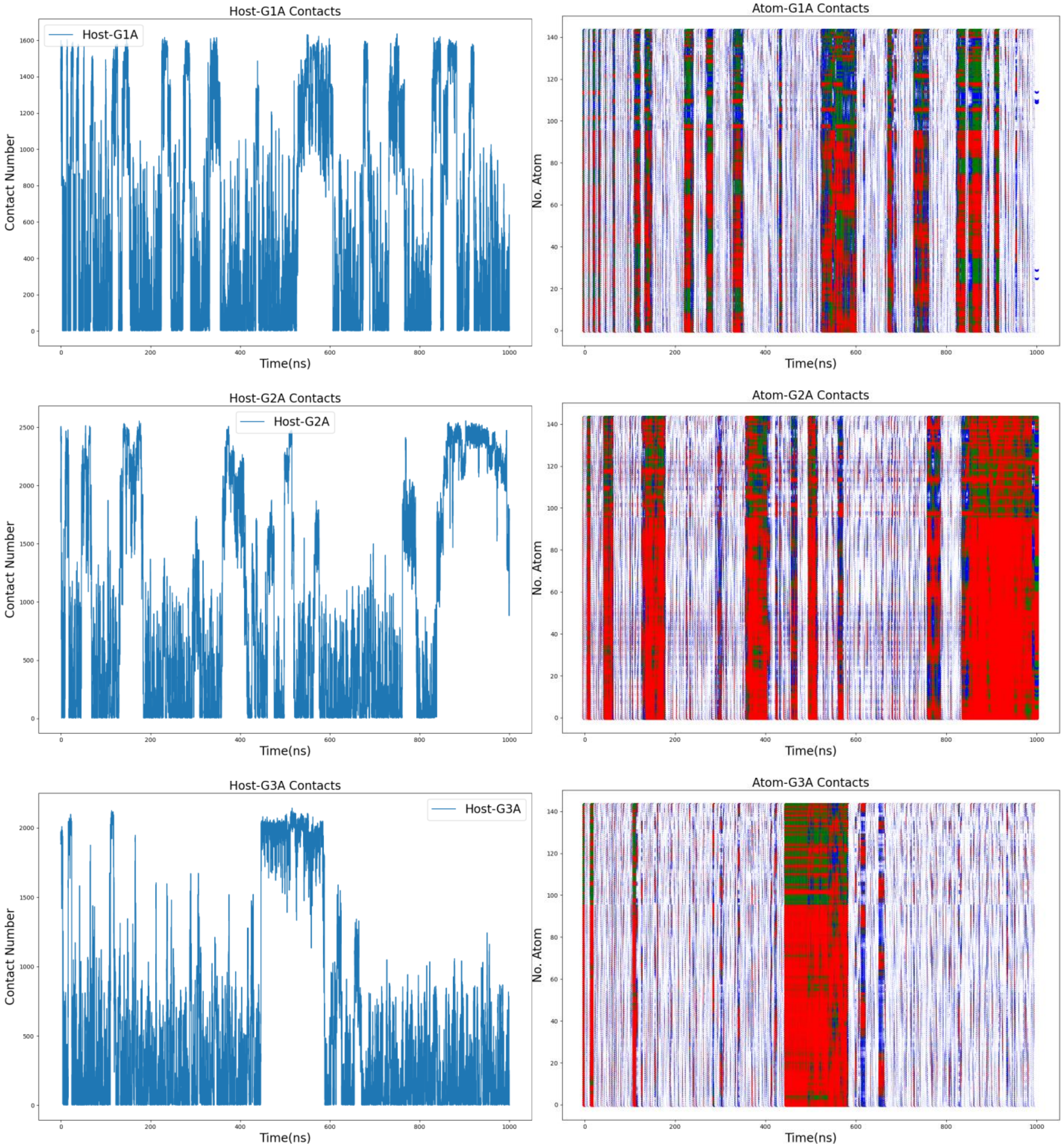

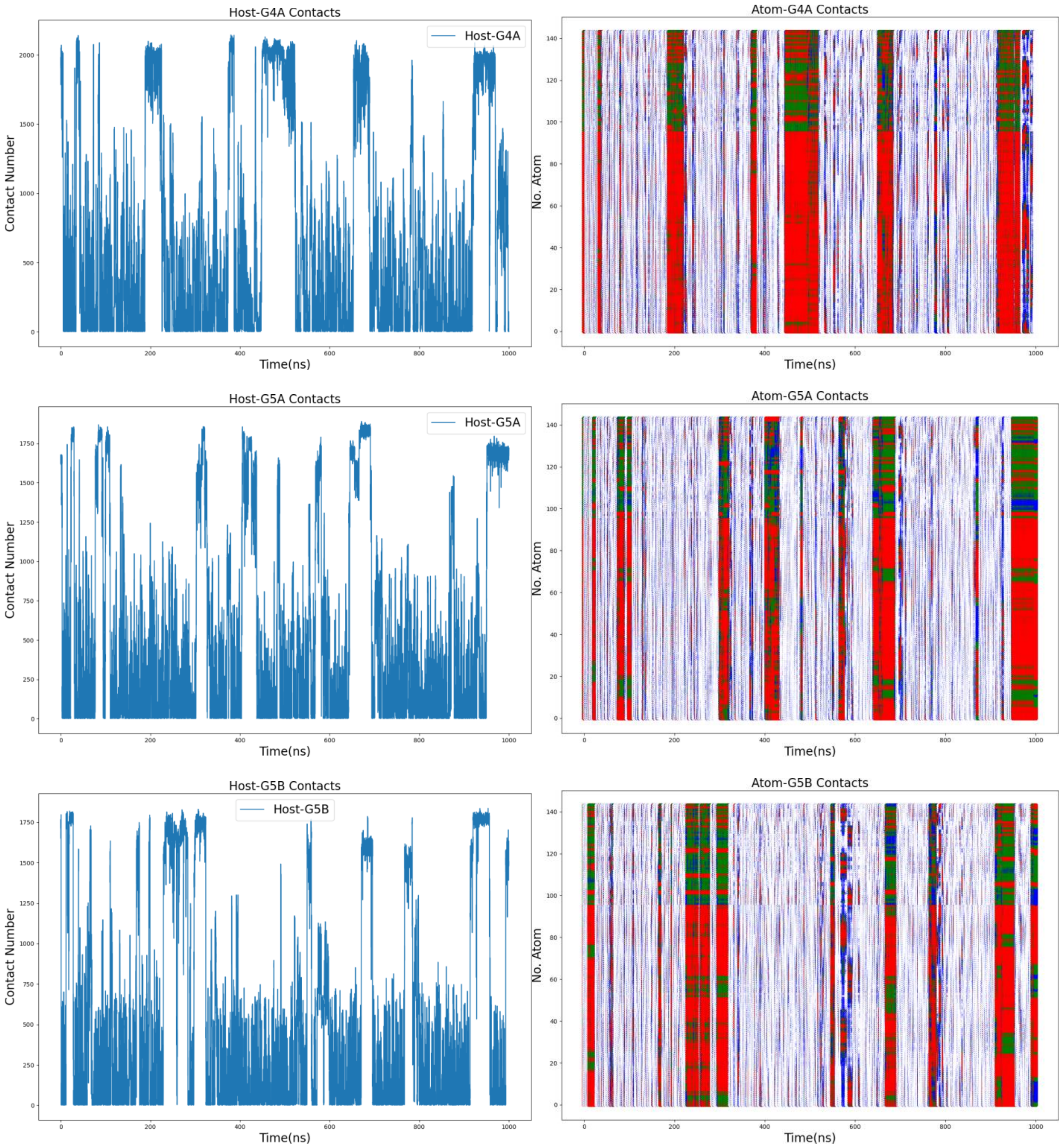
Host-G4A Contacts
Host-G4A
Contact Number
Time(ns)
Atom-G4A Contacts
No. Atom
Time(ns)
Host-G5A Contacts
Host-G5A
Contact Number
Time(ns)
Atom-G5A Contacts
No. Atom
Time(ns)
Host-G5B Contacts
Host-G5B
Contact Number
Time(ns)
Atom-G5B Contacts
No. Atom
Time(ns)

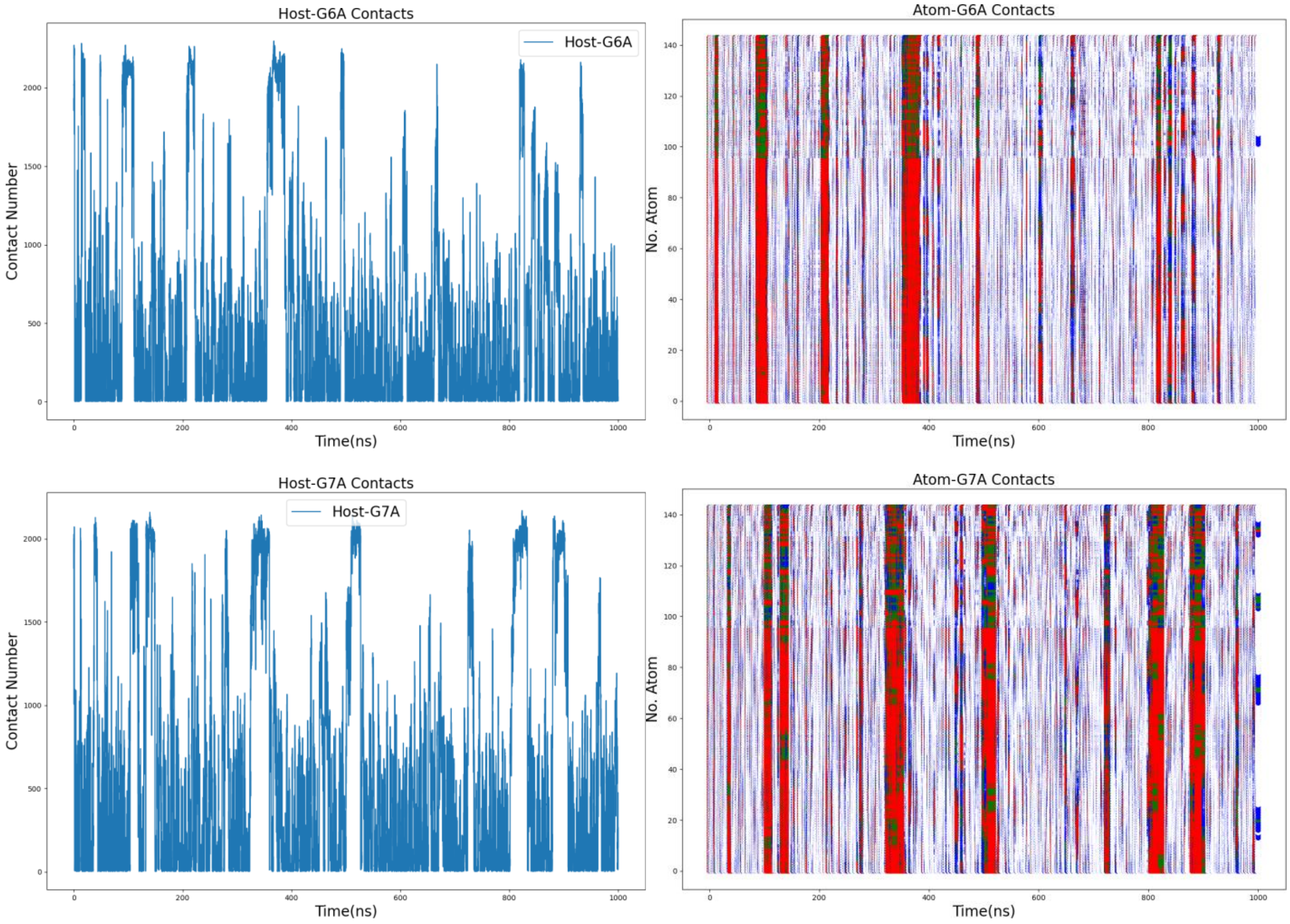
Host-G6A Contacts
Host-G6A
Contact Number
Time(ns)
Atom-G6A Contacts
No. Atom
Time(ns)
Host-G7A Contacts
Host-G7A
Contact Number
Time(ns)
Atom-G7A Contacts
No. Atom
Time(ns)

Fig. S8. The number of contacts between all atoms of the host CB8 and the guests and the by-host-atom decomposition during metadynamics simulations with the FM-BLYP parameter set. The y-axis represents the serial number of host atom. Among the 144 atoms of the host CB8, the first 96 atoms are heavy atoms, and the other 48 atoms are hydrogen atoms. All atoms of the host and the guest are included in the calculation. Red dots denote contacts larger than 10, green dots represent contact number between 5 and 10, blue ones are those larger than 1, and the other are represented by white dots.

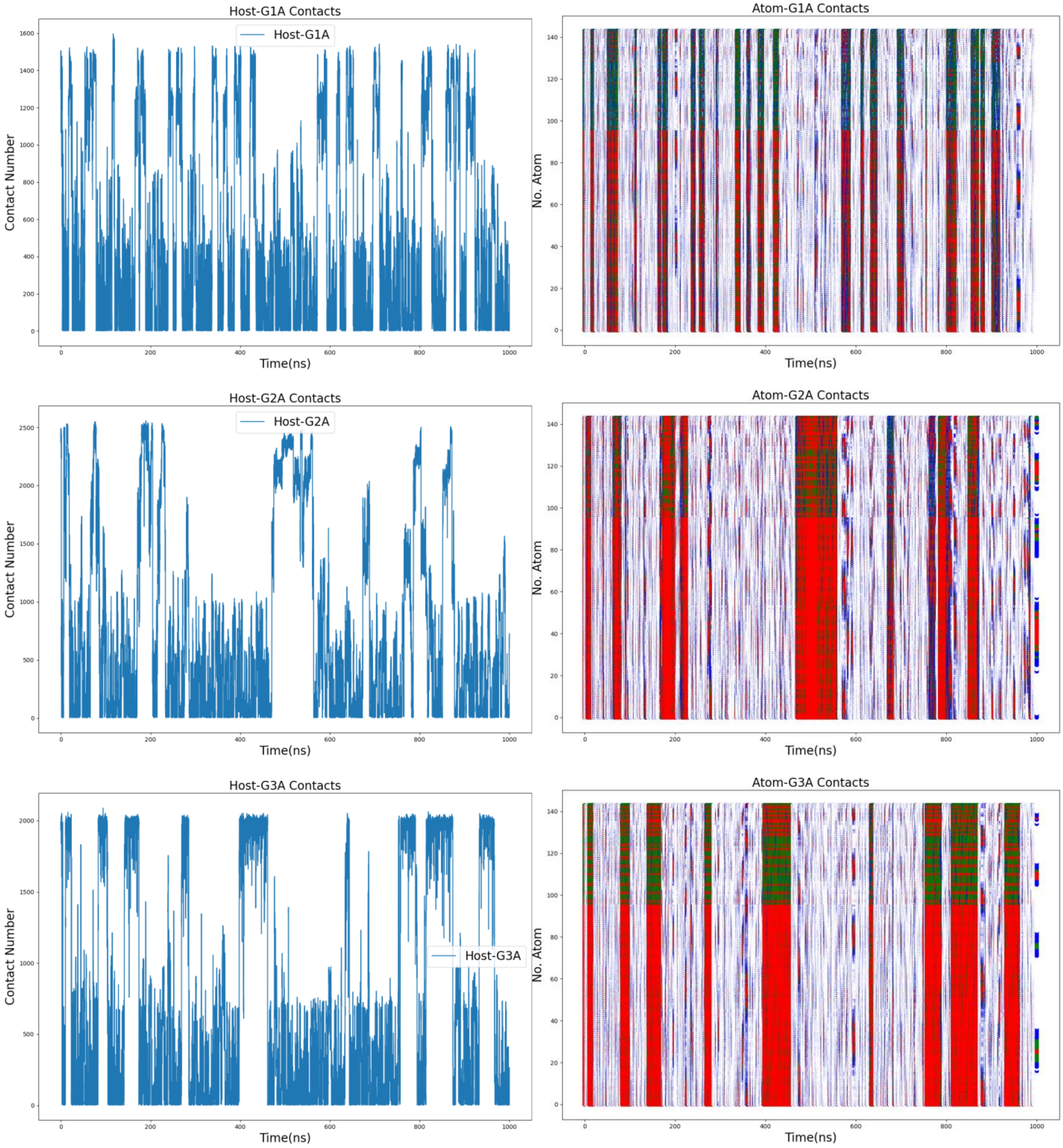

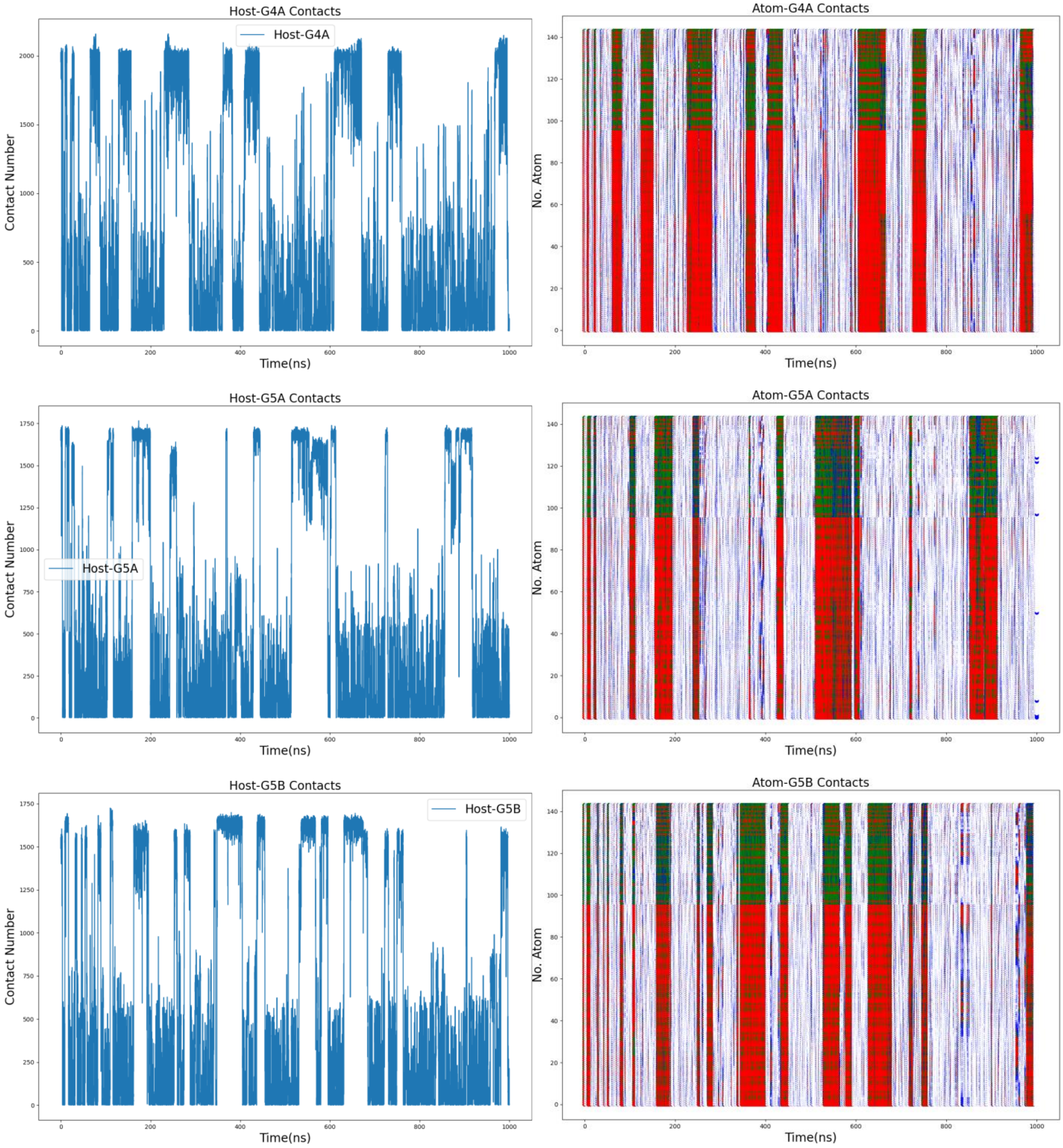
Host-G4A Contacts
Host-G4A
Contact Number
Time(ns)
Atom-G4A Contacts
No. Atom
Time(ns)
Host-G5A Contacts
Host-G5A
Contact Number
Time(ns)
Atom-G5A Contacts
No. Atom
Time(ns)
Host-G5B Contacts
Host-G5B
Contact Number
Time(ns)
Atom-G5B Contacts
No. Atom
Time(ns)

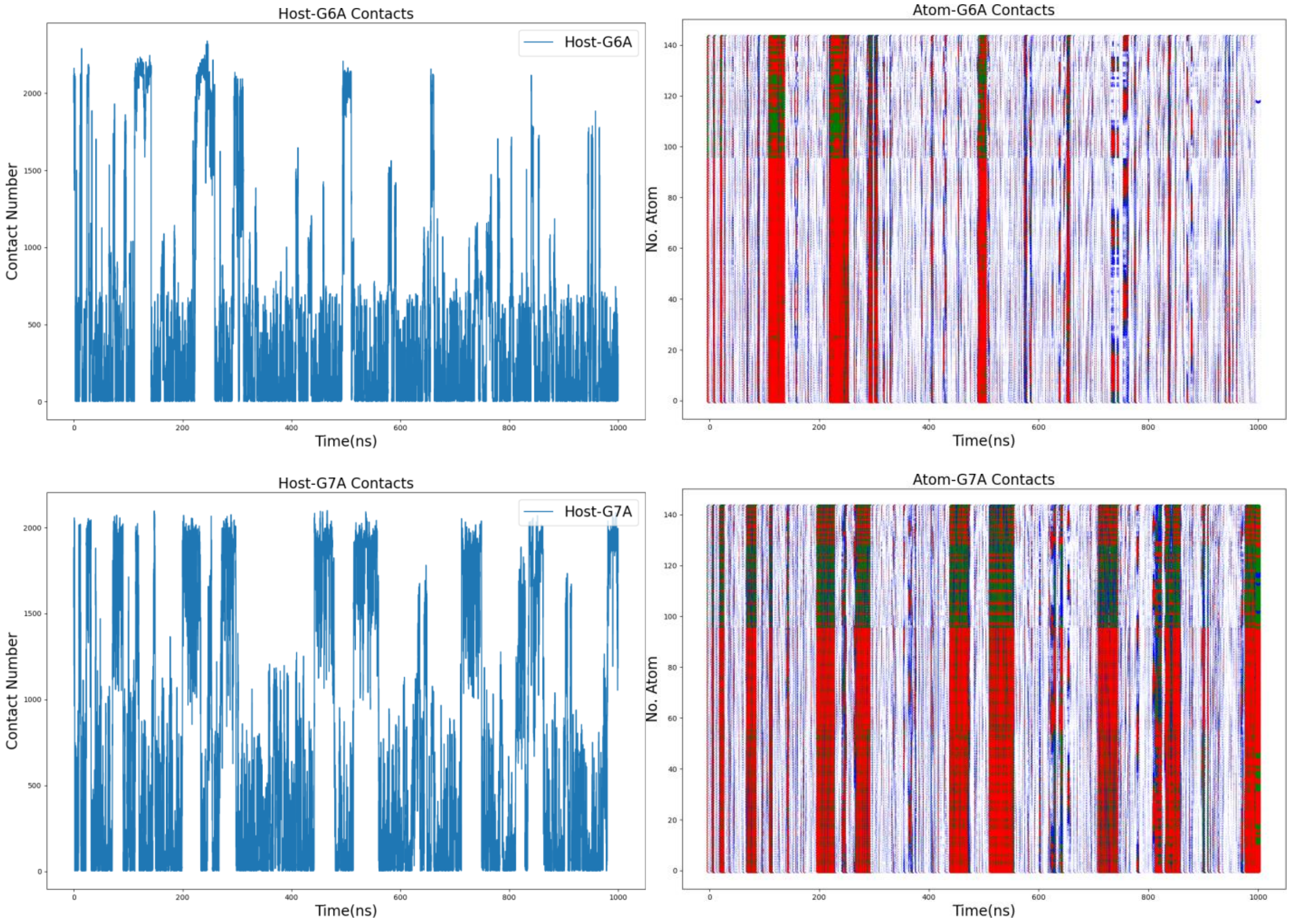
Host-G6A Contacts
Host-G6A
Contact Number
Time(ns)
Atom-G6A Contacts
No. Atom
Time(ns)
Host-G7A Contacts
Host-G7A
Contact Number
Time(ns)
Atom-G7A Contacts
No. Atom
Time(ns)

Fig. S9. 2D $\rho - C$ free energy surfaces in kcal/mol with the GAFF2 force field. Representative structures extracted from the global minimum and the other low-energy local minima are also shown. A worth noting difference between the current GAFF2 binding poses and the GAFF ones reported in our previous work is the closeness or the degree of distortion of the host cavity. As has been shown in the current work, the previously employed GAFF parameter set provides more flexible/softer host ring, which makes it squashed significantly in the bound state. However, the current GAFF2 parameter set describes a stiffer host ring, which is less distorted when coordinating the guest molecules.

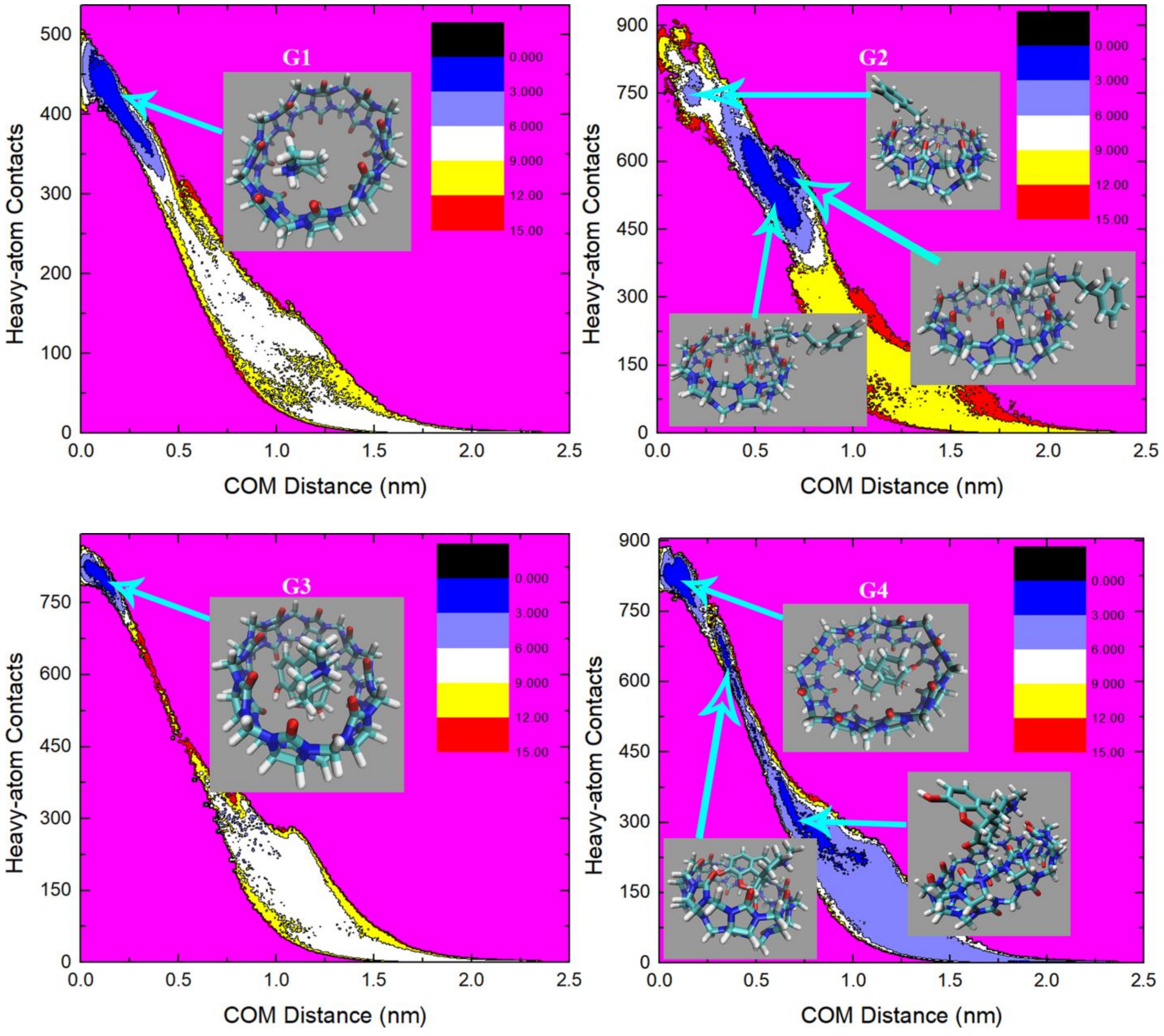

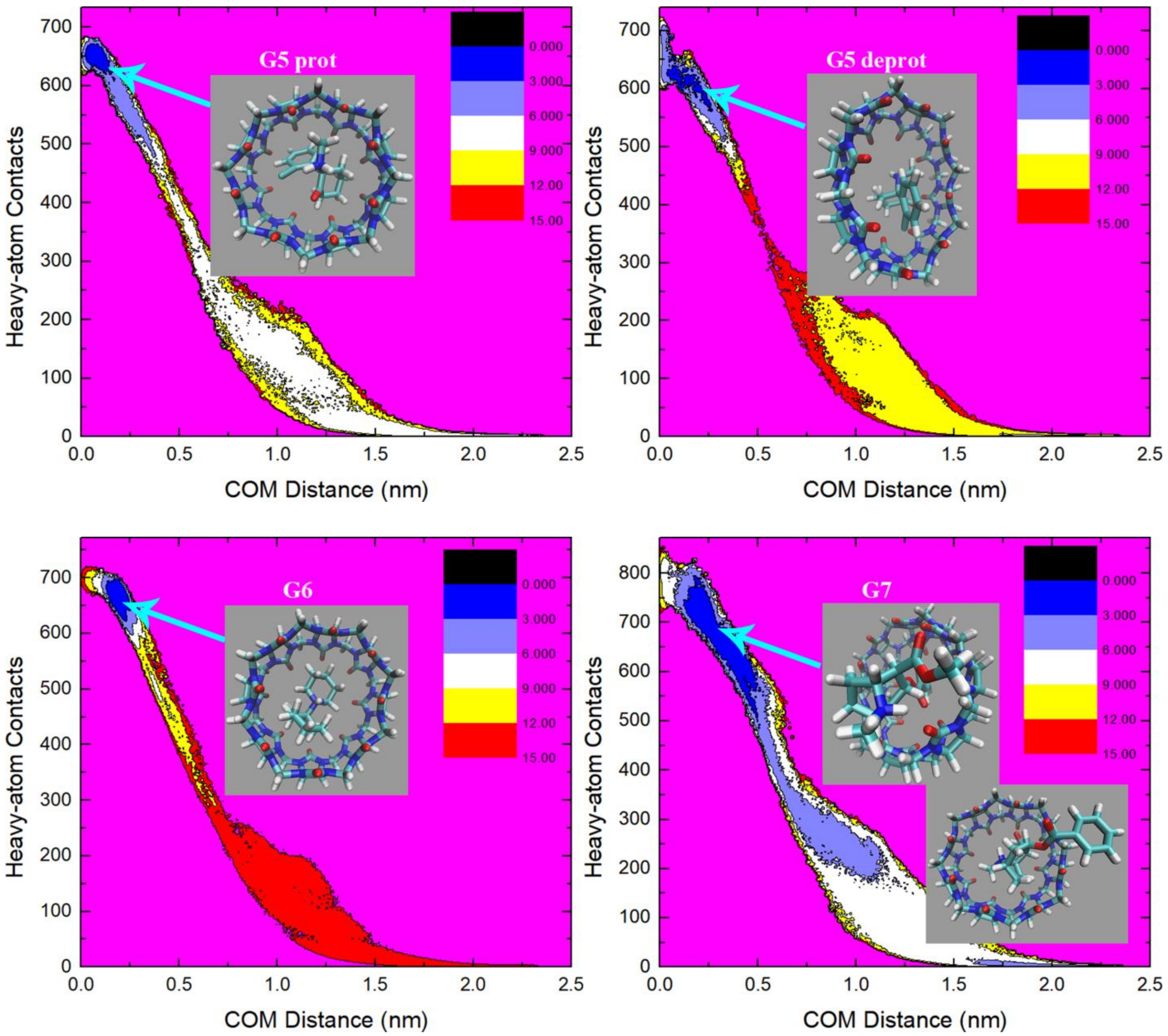
G5 prot
G5 deprot
G6
G7
Heavy-atom Contacts
COM Distance (nm)
0.000
3.000
6.000
9.000
12.00
15.00

Fig. S10. 2D $\rho-C$ free energy surfaces in kcal/mol obtained with the FM-PM6 parameter set. Representative structures extracted from the global minimum and the other low-energy local minima are also shown. Compared with the previous GAFF2 binding modes, those observed under FM-PM6 generally have more flexible and irregularly shaped host cavities, which is in agreement with the observations in unbiased simulations reported in the previous section.

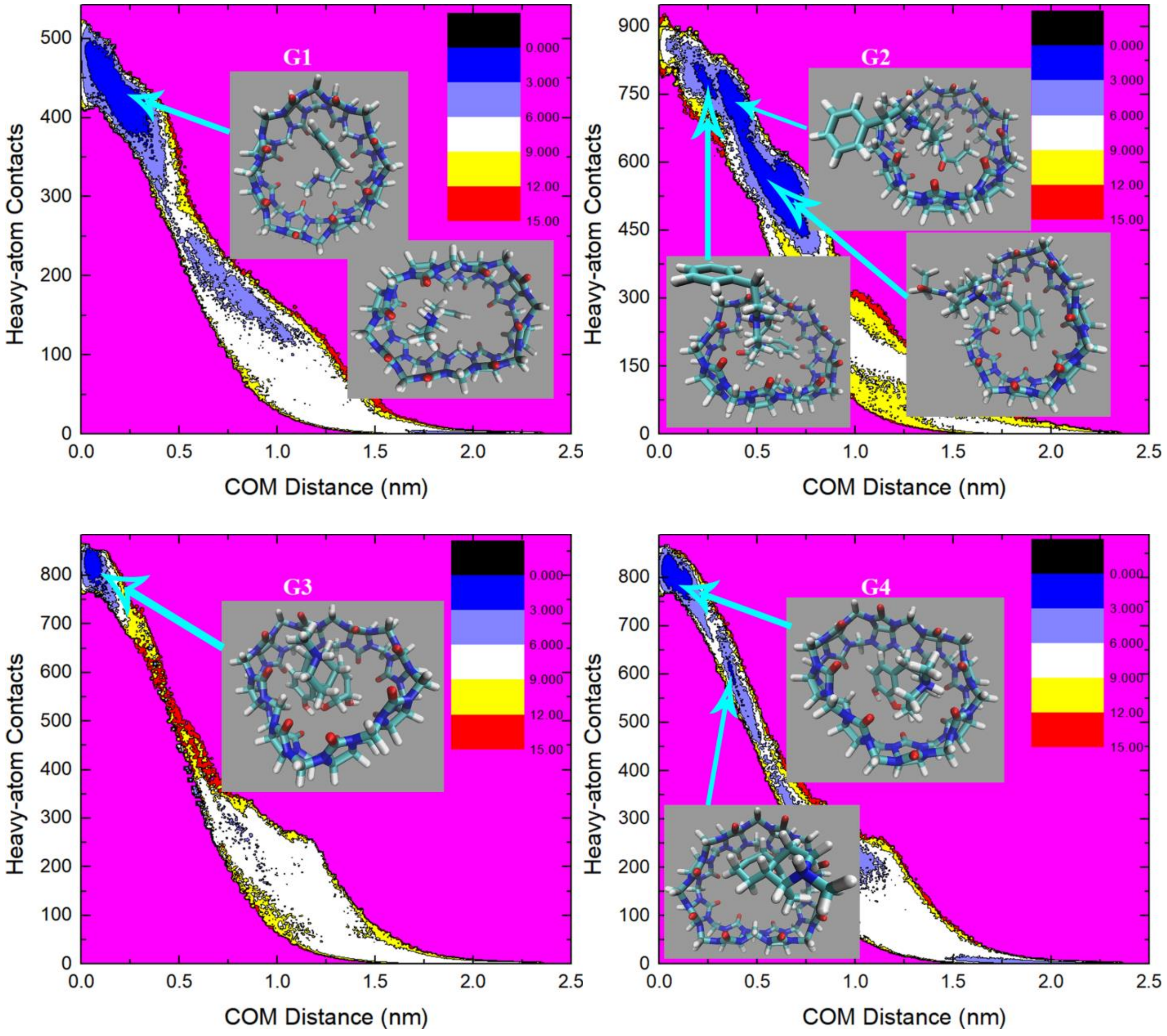

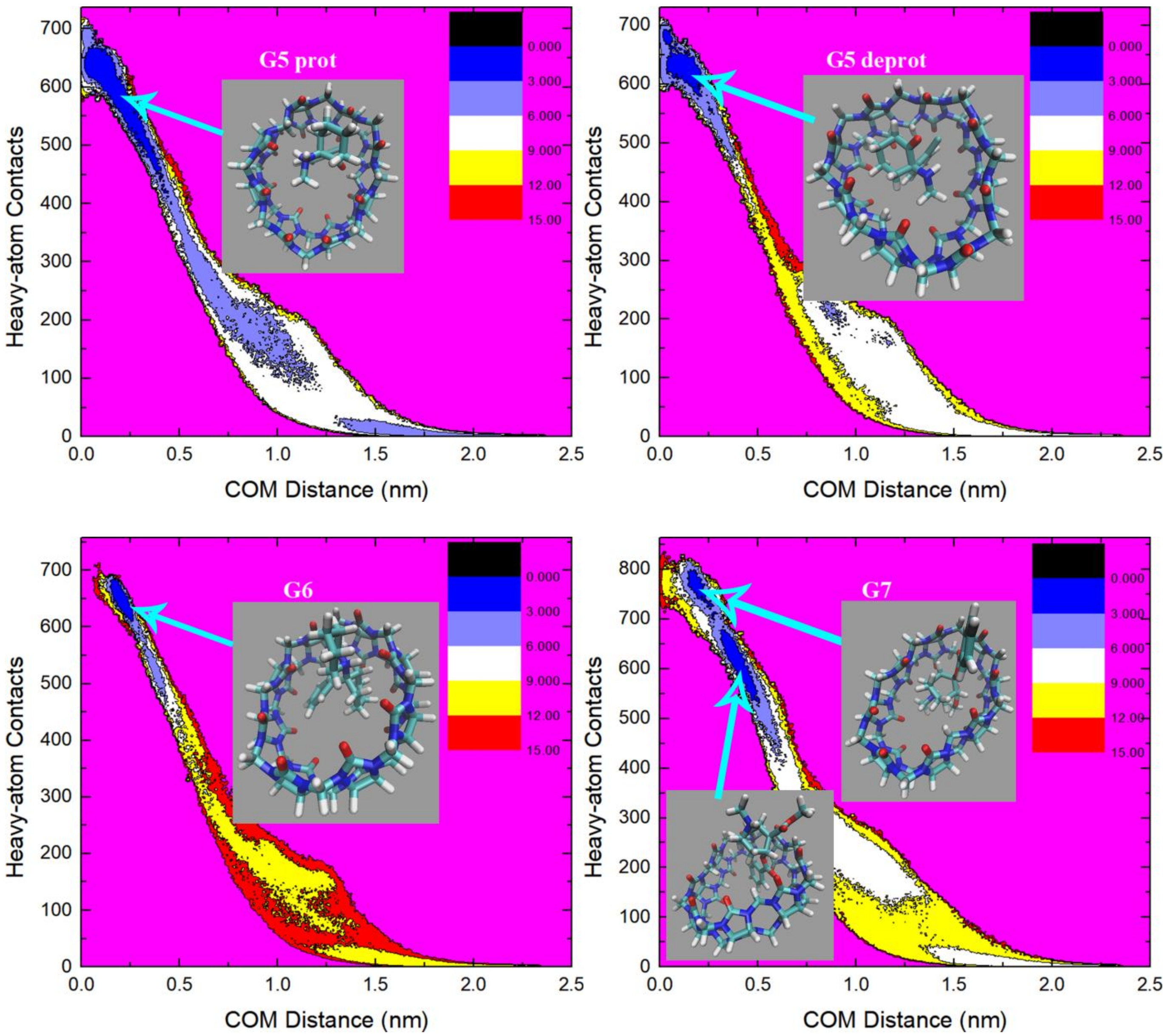
G5 prot
G5 deprot
G6
G7
0.000
3.000
6.000
9.000
12.00
15.00
Heavy-atom Contacts
COM Distance (nm)

Fig. S11. 2D $\rho - C$ free energy surfaces in kcal/mol obtained with the FM-BLYP parameter set. Representative structures extracted from the global minimum and the other low-energy local minima are also shown. The binding poses obtained under the FM-BLYP parameter set are similar to the GAFF2 ones, which agrees with the observations in unbiased simulations.

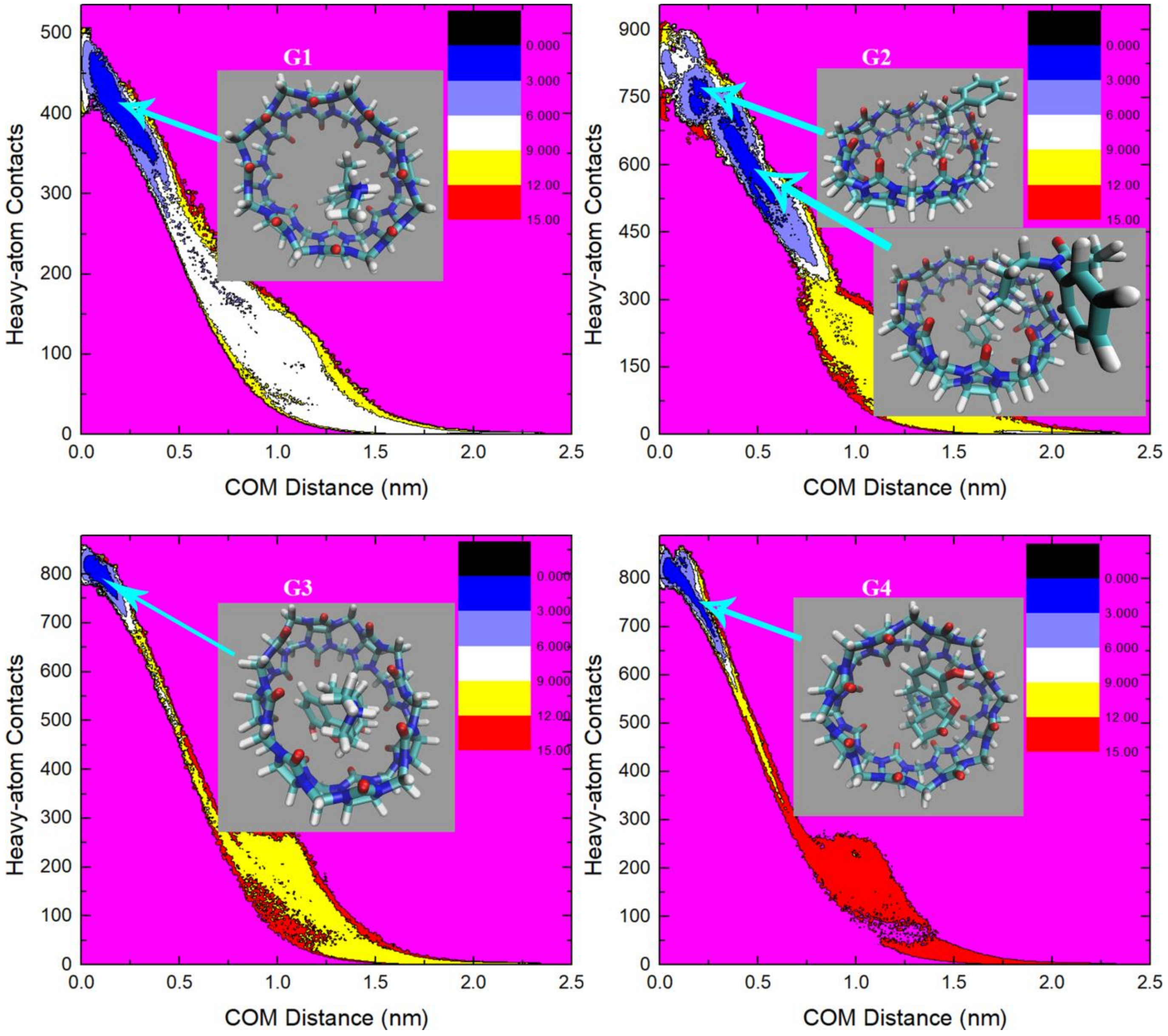

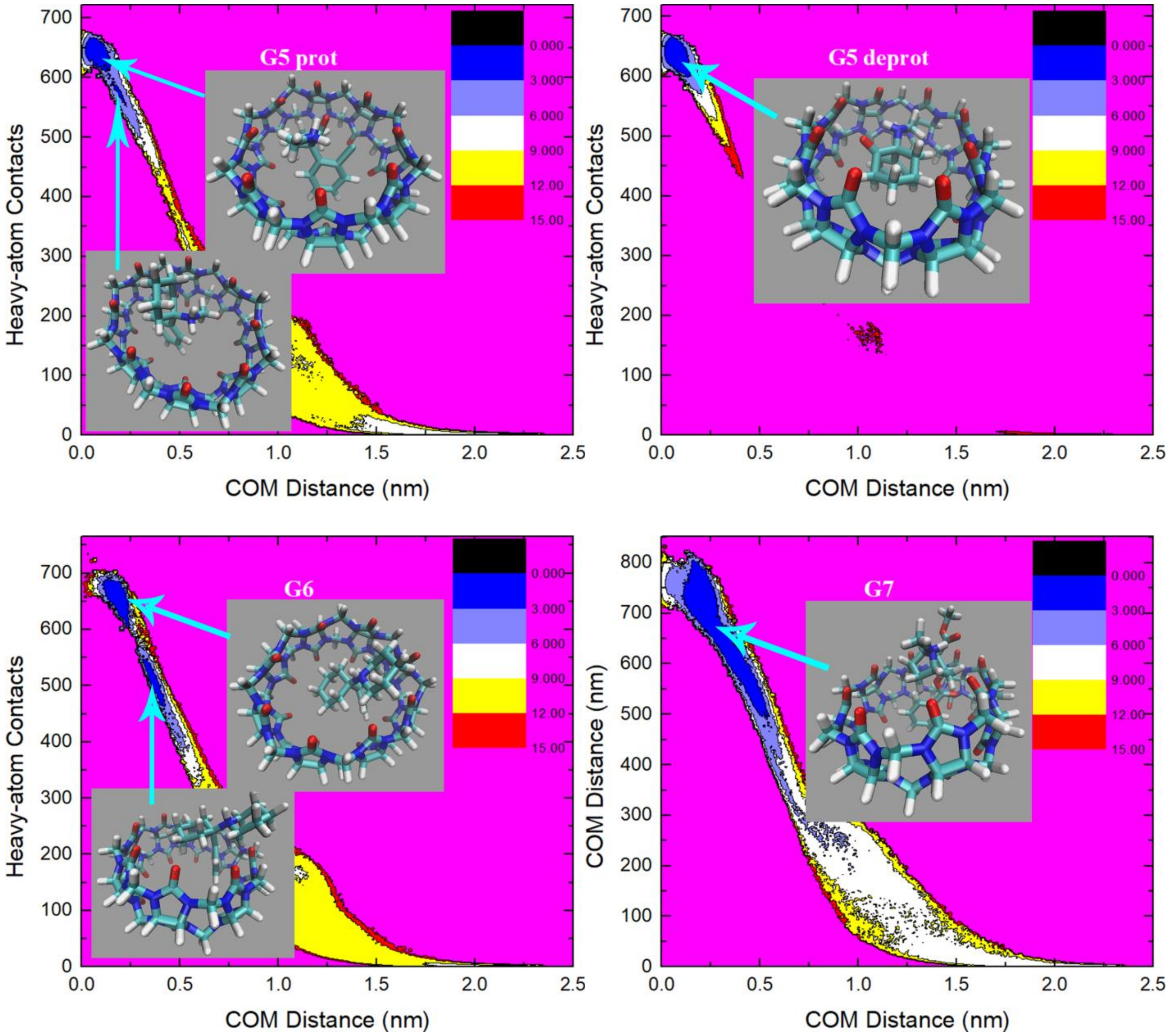
G5 prot
G5 deprot
G6
G7
Heavy-atom Contacts
COM Distance (nm)
0.000
3.000
6.000
9.000
12.00
15.00
